# Adult Human, but Not Rodent, Spermatogonial Stem Cells Retain States with a Foetal-like Signature

**DOI:** 10.3390/cells13090742

**Published:** 2024-04-24

**Authors:** Stephen J. Bush, Rafail Nikola, Seungmin Han, Shinnosuke Suzuki, Shosei Yoshida, Benjamin D. Simons, Anne Goriely

**Affiliations:** 1 MRC Weatherall Institute of Molecular Medicine, Radcliffe Department of Medicine, University of Oxford, Oxford OX3 9DS, UK; 2 Wellcome Trust/Cancer Research UK Gurdon Institute, University of Cambridge, Cambridge CB2 1QN, UK; 3 Division of Germ Cell Biology, National Institute for Basic Biology, National Institutes of Natural Sciences, 5-1 Higashiyama, Myodaiji, Okazaki 444-8787, Japan; 4 Graduate Institute for Advanced Studies, SOKENDAI, 5-1 Higashiyama, Myodaiji, Okazaki 444-8787, Japan; 5 Wellcome—MRC Cambridge Stem Cell Institute, Jeffrey Cheah Biomedical Centre, University of Cambridge, Cambridge CB2 0AW, UK; 6 Department of Applied Mathematics and Theoretical Physics, Centre for Mathematical Science, University of Cambridge, Cambridge CB3 0WA, UK; 7 NIHR Biomedical Research Centre, Oxford OX3 7JX, UK

**Keywords:** germline, single-cell RNA-sequencing, cell cycle, quiescence, cross-species comparison

## Abstract

Spermatogenesis involves a complex process of cellular differentiation maintained by spermatogonial stem cells (SSCs). Being critical to male reproduction, it is generally assumed that spermatogenesis starts and ends in equivalent transcriptional states in related species. Based on single-cell gene expression profiling, it has been proposed that undifferentiated human spermatogonia can be subclassified into four heterogenous subtypes, termed states 0, 0A, 0B, and 1. To increase the resolution of the undifferentiated compartment and trace the origin of the spermatogenic trajectory, we re-analysed the single-cell (sc) RNA-sequencing libraries of 34 post-pubescent human testes to generate an integrated atlas of germ cell differentiation. We then used this atlas to perform comparative analyses of the putative SSC transcriptome both across human development (using 28 foetal and pre-pubertal scRNA-seq libraries) and across species (including data from sheep, pig, buffalo, rhesus and cynomolgus macaque, rat, and mouse). Alongside its detailed characterisation, we show that the transcriptional heterogeneity of the undifferentiated spermatogonial cell compartment varies not only between species but across development. Our findings associate ‘state 0B’ with a suppressive transcriptomic programme that, in adult humans, acts to functionally oppose proliferation and maintain cells in a ready-to-react state. Consistent with this conclusion, we show that human foetal germ cells—which are mitotically arrested—can be characterised solely as state 0B. While germ cells with a state 0B signature are also present in foetal mice (and are likely conserved at this stage throughout mammals), they are not maintained into adulthood. We conjecture that in rodents, the foetal-like state 0B differentiates at birth into the renewing SSC population, whereas in humans it is maintained as a reserve population, supporting testicular homeostasis over a longer reproductive lifespan while reducing mutagenic load. Together, these results suggest that SSCs adopt differing evolutionary strategies across species to ensure fertility and genome integrity over vastly differing life histories and reproductive timeframes.

## 1. Introduction

To maintain sperm production over the reproductive lifespan, spermatogonial stem cells (SSCs) and their progenies regularly undergo multiple rounds of mitotic division. Despite this constant demand, germline mutation rates remain significantly lower than those observed in somatic cells [1]. Although the underlying strategies remain in question, it is thought that they involve DNA replication fidelity and damage [2], and tight regulation of SSC turnover [3,4]. To define the transcriptional signature of germ cell states and their potential conservation across species, emphasis has been placed on the single-cell profiling of whole testes. However, the scarcity and variability of the undifferentiated spermatogonial population and the inherent stochasticity of single-cell transcriptomics data have confounded the detailed characterisation of the stem cell compartment [5,6].

Historically, models of human spermatogenesis date back to the 1960s and posit the existence of two subtypes of type A spermatogonia, A_dark_ and A_pale_, both with undifferentiated nuclear morphology but differential staining intensity with haematoxylin [7,8]. Within this model, only A_pale_ function as actively dividing stem cells [9], while A_dark_ constitute a quiescent population that acts as a regenerative reserve. However, in recent years this ‘hierarchical’ model has been challenged. Through advances in single-cell transcriptomics, studies based on the profiling of whole human testes [10,11] have found evidence of multiple discrete states of type A spermatogonia, none of which unambiguously correlate with an A_dark_ or A_pale_ phenotype [12]. Furthermore, in a previous study applying haematoxylin staining to human seminiferous tubules, many cells were found to have an intermediate morphology, simultaneously resembling both A_pale_ (by staining lightly with haematoxylin) and A_dark_ (possessing a non-staining nuclear rarefaction zone) [13]. There is now an emerging consensus that undifferentiated human spermatogonia can be subclassified into at least four subtypes (reviewed in [14] and based largely on the work of two research groups [15,16]), each stably maintained throughout reproductive life: state 0 (*EGR4*+ *PIWIL4*+ *TSPAN33*+), state 0A (*FGFR3+*), state 0B (*NANOS2*+), and state 1 (*GFRA1*+ *NANOS3*+). Among these, state 0 is considered the most undifferentiated, standing at the apex of the differentiation trajectory.

Contrary to the strictly hierarchical A_dark_/A_pale_ model, the existence of at least four transcriptional states suggests a more flexible and dynamic SSC regulation. In common with mouse spermatogenesis [4,17], it has been proposed that to confer resilience upon the SSC pool and maintain homeostasis over the long reproductive lifespan, humans rely on a pattern of stochastic SSC renewal in which stem cell loss through differentiation is compensated by the duplication of neighbours, leading to a clonal dynamic characterised by neutral drift. Such behaviour would also be compatible with the phenomenon of selfish spermatogonial selection, a process whereby certain pathogenic de novo mutations are transmitted to the next generation at an apparent mutation rate up to 1000-fold higher than background, because they provide a selective advantage to mutant SSCs that clonally expand as men age [18,19].

Despite advances in single-cell transcriptomics, few human testes datasets are available and, accordingly, the molecular identity of the undifferentiated compartments and their regulation remain poorly defined. Specifically, it is still unclear which transcriptional programmes promote SSC development, how SSCs are regulated to maintain lifelong spermatogenesis, and the extent to which these programmes are conserved across species. As spermatogenesis is conserved in mammals, it is also generally assumed that germ cell differentiation starts and ends in equivalent transcriptomic states in related species (although it may not proceed at the same rate through intermediary states) [20]. For instance, a recent study used single-nucleus RNA-sequencing (snRNA-seq) to profile the germline of ten mammals and a bird, characterising spermatogenesis as a common and continuous path of cellular proliferation and differentiation that in each species originated in an analogous, but undissected, SSC cluster [21]. In general, SSCs are underexplored primarily due to the limited number of spermatogonia within the testis [6] and the difficulty in procuring samples.

Here, we hypothesise that an integrated single-cell atlas of the set of publicly available adult human testes samples (comprising data from nine different studies [10,15,16,20,22,23,24,25,26]) would increase the resolution at which we view the molecular identity of the undifferentiated compartment and the origin and progression of the spermatogenic trajectory. Combined with a comparative analysis of spermatogonia across human development (using embryonic, foetal, and both pre- and peri-pubertal samples from seven different studies [10,16,23,25,27,28,29]) and against two primate (rhesus [5,20] and cynomolgus macaque [30]), and five non-primate mammalian species (mouse [31,32,33,34], rat [35], pig [36], sheep [37], and buffalo [38]), here we aim to characterise the heterogeneity of undifferentiated human spermatogonia and provide insight into the nature of SSC populations. In doing so, we propose new hypotheses about the mechanisms of SSC regulation. Specifically, we show that the transcriptional heterogeneity of the undifferentiated compartment varies not only across species but throughout development. Further, based on the projection of a time-series of human single-cell datasets, we suggest a role for the recently characterised state 0B as a suppressive transcriptomic programme that in adults acts to functionally oppose proliferation and maintain SSCs in a mitotically poised or ‘ready-to-react’ state. Supporting this conclusion, we show that foetal germ cells—which are mitotically arrested—share the transcriptional signature of state 0B. Finally, we conjecture that the relative presence and absence of analogous transcriptomic states suggests that the mechanisms of adult spermatogenesis may differ across species. We argue that this divergence reflects differing evolutionary strategies to balance reproductive success with control of the germline mutation rate, ensuring the transmission of high-quality genetic material despite vastly differing life histories and reproductive timeframes.

## 2. Materials and Methods

### 2.1. Integrated scRNA-Seq Atlases of the Post-Pubescent Human Testes and Spermatogonial Stem Cells

To generate an integrated single-cell atlas of human spermatogonial stem cells, we first obtained 34 publicly available post-pubescent (14–66 years) scRNA-seq testes samples, representing data from 29 individuals across 9 studies [10,15,16,20,22,23,24,25,26]. Data sources and sample metadata are detailed in Supplementary Tables S1 and S2, respectively. To mitigate batch effects, we excluded from consideration samples which did not use an Illumina sequencer (e.g., [39]); sequenced too few cells for use with a common informatic workflow, detailed below [11,40,41] (<100 cells; these samples are typically from studies employing the whole-transcript but low-throughput Fluidigm C1/SmartSeq or SmartSeq2 methods [42]); used FACS-sorted, enriched, or otherwise pre-selected cell populations rather than enzymatically digested testes tissue [24,43] (both because the selection process may activate the cells and because the meaningful integration of datasets would not be possible, these samples having biased cell populations); and those where the donor had sex chromosome aneuploidy (e.g., [44]), testosterone-suppression, non-obstructive azoospermia, or otherwise impaired fertility.

For each sample, cell x gene count matrices were obtained using the kallisto/bustools (KB) v0.26.3 ‘count’ workflow [45] with the parameter ‘--filter bustools’ and, as a transcriptomic index, human genome GRCh38.p13, obtained from Ensembl v104 [46] (two indexes were built using this genome, one for expression quantification and one for velocity analysis, discussed below). As an initial QC step for each sample, KB filtered cells on the basis of a knee plot [47], retaining only those above a sample-specific, empirically derived inflection point. For each sample, we also retained only those genes detected in at least 3 cells, and only those cells where the total number of genes was >1000 and <10,000, the total number of UMIs was >2000 and <50,000 and the proportion of mitochondrial genes detected was <5% of the total. Each count matrix was then processed using Seurat v4.0.3 [48] with the normalisation procedure SCTransform [49], setting the method to ‘glmGamPoi’ [50] and mitochondrial gene content, total gene count, total RNA count, and both ‘S score’ and ‘G2M score’ as regression variables (using 67 cell-cycle-phase-associated genes from [51] and listed in Supplementary Table S4). Finally, to produce a testis atlas of 60,427 cells, all 34 samples were integrated using Seurat’s rPCA ‘anchor’ technique with 5000 features. Seurat recommends rPCA for large datasets and requires the user define a ‘mutual neighbourhood’ in which to search for integration anchors. For this purpose, we used as our ‘neighbourhood’ the combined set of 8 samples from the Di Persio [15], Sohni [16], and Zhao [52] studies, as together these contributed the greatest number of cells (Supplementary Table S3)—30,776 cells, or 51% of the total.

To interpret the structure of the testis atlas, transformed gene counts were used for principal component (PC) analysis, with elbow plots (which rank PCs by the percentage of variance explained) used to determine the optimal number of PCs for subsequent analysis, including—for visualisation—performing non-linear dimensional reduction using the UMAP method [53]. The ‘elbow’ was programmatically derived on the basis of being either where the percent change in variation between consecutive PCs was less than 0.1%, or where the PC contributed <5% of the variation and all previous PCs had cumulatively contributed >90%, whichever was lower. We retained for analysis all PCs up to and including this elbow PC.

A k-nearest neighbour graph was then constructed using these top PCs, with community detection (i.e., clustering) performed using the Smart Local Moving algorithm [54] with resolution 0.15. This resolution was empirically chosen on the basis of both a Clustree v0.5.0 [55] dendrogram and manual review (using the interactive browser cellxgene v0.18.0 (https://github.com/chanzuckerberg/cellxgene, accessed on 13 May 2023)), with clusters made at resolutions in the range 0 to 0.5, at intervals of 0.05, interpreted in the context of known testes cell markers (given in Supplementary Table S4 and sourced from [10,16,25,40,41,43,44]) and, to facilitate an unbiased annotation of cluster identities, an all-against-all differential expression analysis.

To that end, we determined the top candidate marker genes per cluster using Seurat’s FindAllMarkers function with parameters min.pct = 0.25, logfc.threshold = 0.25, and only.pos = TRUE, which, respectively, require that a gene be expressed in >25% of the cells in a given cluster, have a log_2_ fold-change difference in expression relative to all other clusters of >0.25 (i.e., have 2-fold greater average expression) and be a positive cluster marker, i.e., be on average more highly expressed in that cluster than all others.

Finally, two clusters of undifferentiated SSCs and differentiating spermatogonia were subset and a higher-resolution ‘SPG atlas’ created by re-running the above QC, SCTransform, and clustering steps on the subset cells. The data were initially clustered at a very high resolution (5.0), such that cluster IDs could be assigned to three small contaminating cell populations; these were removed and the dataset iteratively re-integrated to converge on the final atlas. This process is further detailed within the scripts used for this analysis, available at github.com/sbush/spg_atlas. Note that because meaningful re-integration could not be made for samples with <100 cells and that for any given testis sample, only a small proportion of its cells would be SSCs, the SPG atlas was restricted to data from 9 of the original 34 samples, representing 5 of the 9 studies (data from [10,22,25,26] were excluded).

### 2.2. scRNA-Seq Analysis of Non-Adult Human and Non-Human Samples

Data from 7 foetal and 21 post-natal pre- and peripubertal human scRNA-seq samples (foetal age range 8–23 weeks; post-natal age range 2 days to 13 years) [10,16,23,25,27,28,29], and from 1 pig [36], 1 sheep [37], 2 buffalo [38], 4 cynomologus macaque [30], 11 rhesus macaque [5,20], 8 rat [35], and 37 mouse samples [31,32,33,34], were individually processed using the same KB/Seurat workflow, with the same parameters described above. Data sources and sample metadata are detailed in Supplementary Tables S1 and S2, respectively.

### 2.3. RNA Velocity Analysis

RNA velocity was calculated using scVelo v0.2.5 [56] after having first processed each sample using the KB v0.26.3 ‘count’ workflow [45] with parameters ‘--workflow lamanno --filter bustools --loom’. For each of the 9 samples in the SPG atlas, we then parsed the appropriate ‘counts_unfiltered/adata.loom’ file to extract its list of cell barcodes. We calculated velocity only for cells included in the final SPG atlas, projecting this onto the pre-existing UMAP, embedding using the ‘velocity_embedding_stream’ command.

### 2.4. Enrichment and Impact Analyses

For each cluster in the SPG atlas, GO term and KEGG pathway enrichment was assessed using the R/Bioconductor packages topGO v2.22.0 [57] and SPIA (Signalling Pathway Impact Analysis) v2.46.0 [58], respectively. The topGO package employs the ‘weight’ algorithm to account for the nested structure of the GO tree with correction for multiple hypothesis testing intrinsic to the approach [57]. This requires a reference set of GO terms, built manually from GRCh38.p13/Ensembl v104 and filtered to remove those terms with evidence codes NAS (non-traceable author statement) or ND (no biological data available), and those assigned to fewer than 10 genes in total. Significantly enriched GO terms (*p* < 0.05) were reported only if the observed number exceeded the expected by 2-fold or greater.

To run SPIA, we used the KEGG set of human pathways [59] (available at https://www.genome.jp/kegg-bin/download_htext?htext=br08901.keg, accessed on 8 March 2022). SPIA combines evidence from both a classical enrichment analysis and a bootstrap procedure to quantify the differential impact upon a given pathway. The former considers each pathway a gene list and gives no weight to topology—that is, to the relative position of each gene within that pathway [60]. The latter measures the perturbation on a given pathway by propagating fold changes in expression across its topology. A global probability value, P_G_, is then calculated for each pathway, incorporating the log fold-change of the differentially expressed genes, the statistical significance of the enrichment of any pathway, and the topology of the pathway itself. Consequently, SPIA assesses not only which pathways are enriched for DE genes, but whether the DE is likely to impact upon the function of that pathway. For example, if those DE genes assigned to a pathway are among the downstream genes of that pathway, changes in their expression levels are less likely to affect the pathway as a whole. We retain only those pathways with P_G_ < 0.05 after controlling for an FDR of 5%.

### 2.5. Projection of Individual Samples onto the SPG Atlas

Individual samples, from both human and non-human samples, were projected onto the human whole-testis and SPG atlases using the ‘FindTransferAnchors’ and ‘MapQuery’ commands of Seurat v4.0.3 [48] and following a two-step strategy described below. To facilitate projections, gene names were standardised using one-to-one orthology relationships obtained from Ensembl BioMart [46] release 104 (genome versions GRCm39, mRatBN7.2, GCA_003121395.1, Oar_rambouillet_v1.0, Macaca_fascicularis_6.0, Mmul_10, and Sscrofa11.1, for mouse, rat, buffalo, sheep, cynomolgus macaque, rhesus macaque, and pig, respectively). Cells from a given sample were then projected onto the whole-testis UMAP so as to distinguish germline from somatic cells. Only these putative germ cells were then projected onto the SPG atlas.

Seurat’s projection algorithm works by assigning a mapping score to each cell, from 0 to 1 (lowest to highest), by first projecting the query cell into the reference space, then projecting that cell back onto the query, and then determining to what extent the local neighbourhood was altered in the process (detailed further at https://rdrr.io/cran/Seurat/man/MappingScore.html, accessed 19 February 2023). For human and mouse samples, we required that each cell was projected with a minimum score of 0.8, thereby restricting analysis to cells with high transcriptomic similarity (cells not meeting this threshold would not be shown in the projection figures). For the other species (sheep, pig, buffalo, both macaques, and rat), whose genomes are generally not as well annotated as these models, we varied the projection score from 0 to 0.8 at intervals of 0.2. A projection score of 0 ensured each cell would be plotted according to its ‘best match’ on the SPG atlas, even if in absolute terms that match was poor.

## 3. Results and Discussion

### 3.1. An scRNA-Seq Atlas of Adult Human Spermatogonial Stem Cells

**Figure 1:**
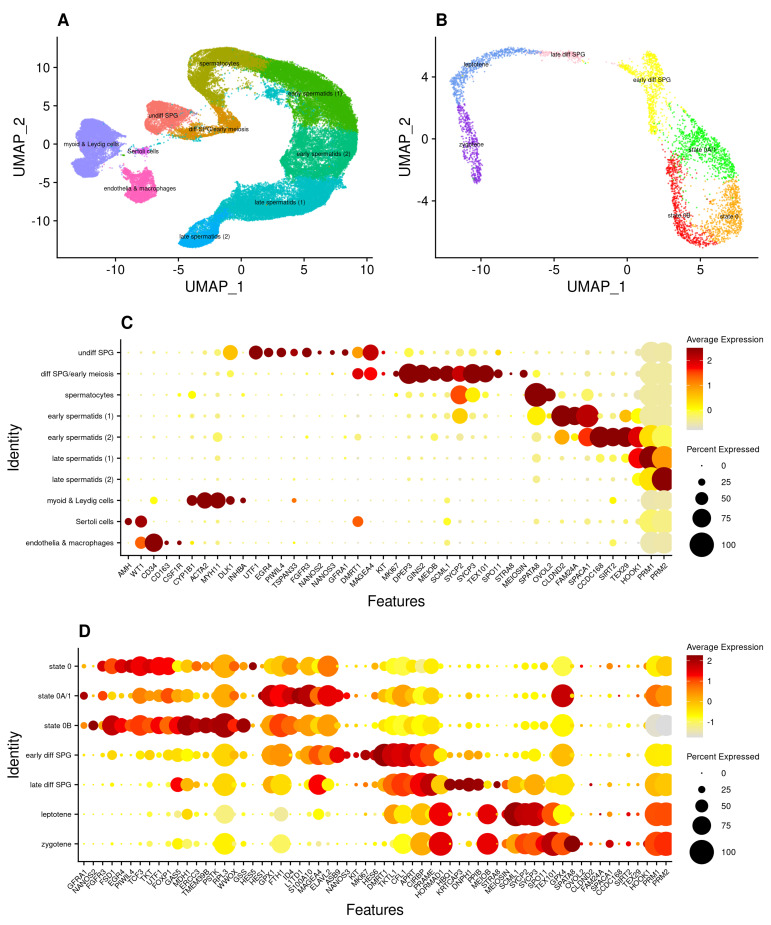
Single-cell expression atlases of (**A**) the adult human testis (n = 60,427 cells), (**B**) a re-clustered subset comprising those cells annotated ‘undifferentiated spermatogonia’ and ‘differentiating spermatogonia’ (n = 4447 cells), hereafter the ‘SPG atlas’. Dot plots showing the relative expression of discriminative biomarkers for each cell type or state are given for both (**C**) the testis atlas, and (**D**) the SPG atlas. Note that the subset of cells comprising the SPG atlas follows a developmental trajectory intentionally broken at the onset of the meiotic programme and that owing to discrete cluster boundaries being imposed upon a continuum of cells, the ‘differentiating spermatogonia’ cluster evidently comprises some primary spermatocytes (i.e., the ‘leptotene’ and ‘zygotene’ clusters of the SPG atlas). Contents of these testis and SPG atlases, showing expression level per gene per cell cluster, are available as Supplementary Tables S5 and S7, respectively. Other versions of the SPG atlas for different clustering resolutions are shown in Supplementary Figure S8 and presented in Supplementary Tables S6 and S8.

To generate an integrated single-cell atlas of human spermatogonial stem cells and their progenies, we obtained 34 post-pubescent (14–66 years) scRNA-seq testes libraries, representing data from 29 individuals across nine previous studies [10,15,16,20,22,23,24,25,26] (detailed in Supplementary Tables S1 and S2). Samples were reprocessed using a common Kallisto/Bustools [45] and Seurat [48] workflow with conservative parameters (see Section 2) to generate an integrated atlas of 60,427 testicular cells (Figure 1), each expressing on average 2877 genes/cell (Supplementary Table S3).

Using an unsupervised clustering approach, we identified 10 distinct cell clusters, which we annotated using both established biomarkers for the major somatic and germ cell types (Figure 1; biomarkers sourced from previous publications [10,16,25,40,41,43,44] and detailed in Supplementary Table S4) and an all-against-all differential gene expression analysis (Supplementary Table S5). Following batch correction (see Section 2), the atlas showed no residual batch effects on the basis of sample accession, study of origin, donor age, or cell cycle phase (Supplementary Figure S1), nor an overtly disproportionate contribution made by any individual sample on the basis of number of genes, mitochondrial genes, or UMIs per cell (Supplementary Figure S2).

The major cell types included undifferentiated spermatogonia (*UTF1*+ *EGR4*+), differentiating spermatogonia (*KIT*+ *MKI67*+), spermatocytes (*SYCP2*+ *SPATA8*+), early spermatids (*SIRT2*+ *TEX29*+), late spermatids (*PRM1*+ *PRM2*+), Sertoli cells (*AMH*+ *WT1*+), endothelial cells (*CD34*+), peritubular myoid cells (*ACTA2*+ *MYH11*+), Leydig cells (*DLK1*+ *INHBA*+), and macrophages (*CD163*+ *CSF1R*+) (Figure 1 and Supplementary Table S5). Sertoli cells comprised the smallest cluster, consistent with the fact that they are especially sensitive to digestion and size selection—essential steps within many of the respective scRNA-seq protocols [61].

As our primary focus was to resolve germ cell rather than somatic cell types, we empirically selected the minimum clustering resolution necessary to relate the germ cell clusters to established cell types, rather than risk over-clustering and segregating cells on the basis of non-biological variation. While this approach maximises the inclusion of undifferentiated spermatogonia in a single cluster (for subsequent refinement, discussed below), this approach may under-cluster some of the somatic cell types and mask their underlying biological structure. To that end, myoid and Leydig cells were aggregated into one supercluster, as were endothelial cells and macrophages (Figure 1). As an independent validation of data integrity, reinforcing the annotation of the two spermatogonial clusters (‘undiff SPG’ and ‘diff SPG’), we found that of a list of 43 genes previously detected only in human spermatogonia at the protein level, 23 (54%) were found to be differentially expressed only in either of these two clusters and no other germ cell cluster (specifically, *CBL*, *CD9*, *CHEK2*, *DMRT1*, *DMRTB1*, *ELAVL2*, *EPCAM*, *EXOSC10*, *FGFR3*, *FMR1*, *ID4*, *ITGA6*, *ITGB1*, *MAGEA4*, *PASD1*, *PHF13*, *POU2F2*, *SPOCD1*, *SSX3*, *TRAPPC6A*, *UCHL1*, *UTF1*, and *ZBTB16*) [12].

These two clusters of (predominantly) pre-meiotic germ cells were extracted and re-integrated to create an adult ‘SPG atlas’ of 4447 cells (Figure 1), which we also re-clustered using an unsupervised approach. As with the whole-testis dataset, we found no evidence of batch effects (Supplementary Figure S3) nor disproportionate contribution by individual samples (Supplementary Figure S4). However, as spermatogonia constitute a small proportion of the total number of testicular cells and their meaningful integration could not be made for samples with <100 cells (as integration requires the identification of comparable cell populations across multiple samples), the atlas was restricted to data from nine (26%) of the original 34 libraries, representing five of the nine original studies (more specifically, data from refs. [10,22,25,26] were excluded). Nevertheless, each contributing sample and each study made an approximately proportionate contribution of UMIs to each of the spermatogonial clusters, such that downstream analyses were unlikely to be influenced by residual sample- or study-specific effects (Supplementary Figure S5). Reciprocal projection of the SPG atlas onto the whole-testis atlas, and of the ‘undiff SPG’ and ‘diff SPG’ clusters of the whole-testis atlas onto the SPG atlas, further confirmed that the two atlases shared the same cell populations (Supplementary Figure S6). The expression of key marker genes is shown in Supplementary Figure S7 and used to inform annotation, discussed below.

To further develop our analysis, we then sought to subtype the undifferentiated compartment (depicted as the ring-like structure in Figure 1B), noting that the number of cell clusters within this population is algorithmically derived, a function of Seurat’s ‘cluster resolution’ parameter. We first used Clustree [55] to visualize the effect of different resolutions, finding the number of substates varied stepwise from two to four at resolutions 0.3, 1.1, and 1.2, respectively (see Supplementary Figures S8 and S9, with the respective datasets available as Supplementary Tables S6–S8). For subsequent analysis, we clustered the atlas at resolution 1.1 on the basis that it split the ring into three distinct clusters that could each be related to the existing literature (Figure 1), discussed more fully below.

At this resolution, the SPG atlas comprised seven distinct clusters, representing both undifferentiated states and distinct differentiating cell types, which we annotated on the basis of established biomarkers (Supplementary Table S4) and global differential gene expression analysis (Supplementary Table S8). To attach meaningful names to each cluster, we noted that three recent studies (Di Persio [15], Sohni [16], and Guo [10]) reinforced each other by defining analogous undifferentiated cell states, albeit with varying nomenclature (Supplementary Table S4). We adopted the terminology of Guo [10] and Di Persio [15], and annotated the three undifferentiated cell clusters as ‘state 0’ (*EGR4*+ *PIWIL4*+), a combined ‘state 0A/1’ (*FGFR3*+ *GFRA1*+), and ‘state 0B’ (*NANOS2*+), respectively, with the remaining four clusters of differentiating cells annotated as ‘early differentiating spermatogonia’ (early diff SPG; *DMRT1*+ *KIT*+ *MKI67*+), ‘late differentiating spermatogonia’ (late diff SPG; *STRA8*+), ‘leptotene’ (*SYCP3*+), and ‘zygotene’ (*SPATA8*+).

Note that although the same dataset produced two undifferentiated cell clusters from resolutions 0.3 to 0.6, three from 0.7 to 1.1, and four from 1.2 to 2.0 (we did not continue clustering at higher levels), the cells on the right-hand side of the ring formed two stable clusters from resolution 0.7 onwards, and those on the left-hand side formed either one (resolutions 0.7 to 1.1) or two (resolution 1.2 and higher) clusters, respectively. Nevertheless, without foreknowledge of the biological significance of these putative undifferentiated states, it is difficult to determine whether the cells have been under- or over-clustered at a given resolution [62]. Under-clustering masks biological structure by assigning cells to clusters that are too broad, whereas over-clustering segregates cells on the basis of non-biological variation. In this respect, resolution 1.1 may be considered ‘optimally’ clustered, as there were no apparent noisy or artefactual populations, and each of its clusters could be related to known cell states without defining new ones (i.e., the two subtypes of state 0B, which formed only at resolution 1.2 and higher). However, we noted no clear distinction between state 0A and state 1, which were considered separate *FGFR3*+ and *GFRA1*+ *NANOS3*+ populations by [15] (in our SPG atlas, *FGFR3*, and *GFRA1* are differentially expressed in the same undiff SPG cluster, whilst *NANOS3*+ is differentially expressed in ‘early diff SPG’; Supplementary Table S7). Regardless, it is important not to lose sight of the fact that these are discrete states imposed on what in reality is a continuum, with some studies suggesting a gradual transition between states rather than binary on/off programmes [10,15,24,41]. Indeed, consistent with this, there were very strong correlations between the expression profiles of each of the three undifferentiated cell clusters, which primarily differed not in the identity of the genes they expressed, but in their relative levels of expression (Supplementary Figure S10).

Of the 4447 cells in the germ cell atlas, 2229 (50%) were undifferentiated (i.e., the total number of cells annotated as states 0, 0A/1, and 0B, collectively forming the Figure 1B ring). Note that in their respective UMAPs, the studies by Sohni [16] and Di Persio [15] also identified this ring-like structure and noted the differential enrichment of *FGFR3* and *NANOS2* on opposite sides (as also seen in Supplementary Figure S7). However, neither study speculated in much detail as to its origin or biological significance. Here, using the larger integrated datasets, we aimed to refine and expand upon these findings.

### 3.2. Subpopulations of Undifferentiated Spermatogonia with Differing Metabolic Profiles

At the chosen level of clustering (resolution 1.1), the three subpopulations of undifferentiated spermatogonia segregated into states at the bottom (state 0), right (state 0A/1), and left (state 0B) of the ring-like structure shown in Figure 1B. The cell population immediately above the ring constitutes differentiating spermatogonia, with the apparent gap between this cluster and the onset of the meiotic programme likely reflecting both the conservative thresholds employed to generate the atlas and the capture of few cells at this stage of differentiation.

To characterize the nature of the undifferentiated subpopulations, we first applied RNA velocity analysis, a method of inferring cell fate trajectories from the relative abundance of nascent (unspliced) and mature (spliced) mRNA [56], illustrated in Supplementary Figure S11. For the cells within the ring, we made three observations. First, cells in state 0 appeared to be, for the most part, transcriptionally ‘undirected’, consistent with their potential identity as a ‘reserve’ population and the starting point (i.e., apex) of the spermatogonial trajectory. Second, vectors aggregated at the very top of the ring, in state 0A/1, with arrows oriented upwards from both state 0A and state 0B, and downwards from ‘early diff SPG’. Third, within state 0B, there were two sets of streamlines, one pointing towards state 0A/1 and another towards state 0 (note that at resolution 1.2, the state 0B cluster splits into two on this basis; see Supplementary Figure S9). Velocity plots are characteristically challenging to interpret, although taken together these observations suggest that undifferentiated spermatogonia could be transcriptionally orientated both towards and away from state 0, such that the spermatogonial trajectory may not proceed linearly from a state 0 ‘start’. Rather, the transcriptomic programmes of 0A and 0B may be interpreted as, in part, directed towards and away from a commitment to differentiation, respectively.

To explore this concept further, we performed GO and KEGG enrichment analysis on the set of genes differentially expressed (DE) in each cluster, using both all-against-all and pairwise comparisons (Supplementary Tables S10–S12). Consistent with the energetic expense of differentiation, cells in state 0A/1 had a metabolic profile shifted towards oxidative phosphorylation (OXPHOS). GO terms enriched among genes DE in state 0A/1, relative to the other six clusters in the atlas (i.e., including undifferentiated and differentiating spermatogonia, and cells at the onset of meiosis), included ‘aerobic respiration’, ‘ATP metabolic process’, and ‘mitochondrial respiratory chain complex assembly’ (Supplementary Table S10). Furthermore, a pairwise analysis of genes differentially expressed in state 0A/1 relative only to either states 0 or 0B showed enrichment for essentially the same terms, including ‘mitochondrial ATP synthesis coupled electron transport’ and ‘aerobic electron transport chain’ (Supplementary Table S11). The implication is that spermatogonia in the other undifferentiated states have a relatively hypoxic metabolic profile, with cells in state 0A having transitioned away from this. Also consistent with this, DE genes in state 0A/1 relative to state 0 were enriched for the term ‘regulation of transcription from RNA polymerase II promoter in response to hypoxia’ (Supplementary Table S11). We note, however, that there was no direct evidence of enriched GO or KEGG terms related to (for example) glucose metabolism, and that a condition of relative hypoxia in states 0 and 0B is inferred. Conversely, however, we found that both *ECSIT*, a regulator of the balance between mitochondrial respiration and glycolysis [63], and *PGLS*, a component of the pentose phosphate pathway (PPP), were differentially upregulated in state 0B (Supplementary Table S7). The latter is of interest because PPP enzymes are downregulated in hypoxic conditions, facilitating the shift in glucose metabolism towards direct glycolysis [64] (see also Supplementary Text for additional evidence of glycolysis in state 0, using an orthogonal dataset [38]). As state 0 appeared less inclined towards OXPHOS (and thereby differentiation), we characterized this cluster in further detail.

### 3.3. The ‘State 0’ or ‘SSC Reserve’ Cluster Has Little Transcriptomic Evidence of G_0_ Quiescence

Quiescence (defined as reversible growth arrest) is a common, but not requisite, feature of many stem cell populations [65], including neural [66] and hematopoietic [67], which reside in hypoxic niches and rely on glycolytic metabolism. In the classical hierarchical model of spermatogenesis, A_dark_ are thought to constitute a quiescent SSC reserve, replenishing the pool of actively cycling SSCs (i.e., those associated with state 0A/1, or A_pale_ in the classical model). However, on the basis of the single-cell atlas, and irrespective of any inference of hypoxia, there is no compelling evidence that as a whole ‘state 0’ is quiescent. To draw this conclusion, we considered cell cycle scoring, GO/KEGG enrichment in state 0, and the relative expression of established G_0_-markers in better-characterised stem cell systems.

First, based on their transcriptional signature, we found that the majority of undifferentiated spermatogonia were predicted to be positioned in either the S or G_2_/M phases of the cycle, i.e., actively replicating DNA, with very few cells in G_1_ (and, by implication, G_0_). Of the 725 cells in the state 0 cluster, only 90 (12%) were scored as G_1_ (Supplementary Table S13). Similar proportions have been reported in mouse germ cells, in which 3% of undifferentiated spermatogonia were predicted to be in G_1_ phase and the remainder actively dividing (43% in S phase and 55% in G2/M) [52]. Second, GO term enrichment analysis indicated a relatively high degree of cellular activity. The set of genes differentially expressed in state 0 were enriched for ‘cytoplasmic translation’, with 40% of the 159 genes annotated with this term differentially expressed in state 0 (Supplementary Table S10). Third, of a set of 101 genes, 68 of which comprised a quiescent gene signature from a previous study [68] using microarrays to identify genes consistently up- or downregulated in quiescent haematopoietic stem cells (HSCs), muscle stem cells and hair follicle stem cells, and 35 of which were considered either positive or negative intrinsic regulators of HSC quiescence (in either humans [69] or mice [70]), only nine were differentially expressed in state 0 and only three exclusively in state 0 (Supplementary Table S14). Similarly, there was neither differential expression of *GPRC5C*, considered a marker for a ‘highly dormant’ subpopulation of human HSCs [71], nor *DNMT3L* [72], an epigenetic regulator involved in the promotion of quiescence in postnatal mouse SSCs, the latter showing detectable expression in just 1.1% of undifferentiated cells (Supplementary Table S7).

Further supporting the comparatively high transcriptional activity of state 0, enriched GO terms for genes differentially expressed in state 0 relative to state 0A/1 include ‘histone H4 acetylation’ (associated with the opening of chromatin) and ‘positive regulation of histone H3-K4 methylation’ (Supplementary Table S11). Of note, H3-K4 methylation mitigates transcription–replication conflicts (TRCs) during periods of high transcription by decelerating replication rate [73]. In the context of a lower germline mutation rate, this is notable since TRCs, which arise when the transcription and replication machinery operate simultaneously on the same DNA template, are inherently mutagenic. Interestingly, there were no GO terms substantively enriched among the set of genes upregulated in state 0 relative to 0B. While there were 10 significantly enriched GO terms in this pairwise comparison, no term was supported by more than seven differentially expressed genes; Supplementary Table S11). This lack of relatively distinct biological activity is suggestive of either a high degree of similarity between states 0 and 0B, or of state 0 being an ‘undirected’ state to which a subset of cells in state 0B transition (as also implied by the RNA velocity plot).

While cells in the state 0 cluster exist in a relatively hypoxic state, before the metabolic shift to OXPHOS that precedes cellular maturation and differentiation, our overall interpretation is that—contrary to what has been described before [10]—they are unlikely to be G_0_-quiescent. Nevertheless, it remains possible that a small and undistinguished subset of cells may still be quiescent. This would reconcile the observations of this cluster’s lack of direction on the velocity plot, its small number of ‘quiescent signature’ genes (n = 9; Supplementary Table S14), its relative enrichment for translational activity (a possible explanation for which is that it primarily contains cells captured immediately after they exited quiescence), and the findings of both a single-cell lineage-tracing study [74], which posits a quiescent SSC population marked by *FOXC2*, and a histological study [75], which posits a small subpopulation of undifferentiated spermatogonia as a quiescent reserve, A_dVac_ (‘A_dark_ with nuclear rarefaction zone’)—potentially too few in number to have been clustered separately here. We cannot rule out the possibility that we did not directly detect an obviously quiescent pool of cells simply because current implementations of scRNA-seq are not able to capture either enough cells, or enough of the transcriptome of these cells, to meaningfully distinguish them. To that end, it is worth recalling that the data used to construct the atlas comprise unselected/unsorted cells, largely from biopsies.

### 3.4. The ‘State 0B’ Transcriptional Programme Is Characteristic of Foetal Germ Cells, Likely Suppresses Cell Proliferation, and Is Retained into Adulthood in Humans

**Figure 2:**
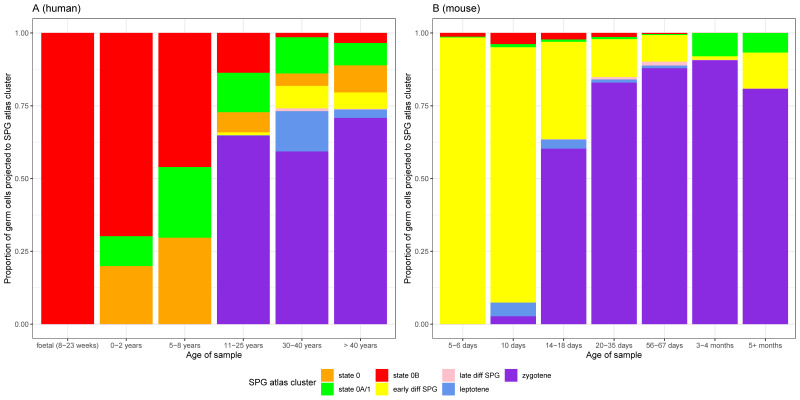
The relative composition of the undifferentiated spermatogonial pool varies throughout development and across species. Bars show the proportion of germ cells from human (**A**) or mouse (**B**) at different ages (X-axis), projecting onto each of the seven clusters/states of the SPG atlas (shown in Figure 1B), represented by different colours. (**A**) Projection of individual human samples onto the SPG atlas (n = 62, representing 6 age groups) is consistent with the maintenance of foetal primordial germ cells in mitotic arrest, their progressive postnatal re-entry into the cell cycle, and the onset of meiosis at puberty. That mitotically arrested cells exclusively project to state 0B (red) suggests a function in suppressing cell proliferation. (**B**) Projection of individual mouse samples (n = 37, representing 7 age groups) shows a comparatively rapid onset of the meiotic programme and the loss of state 0B after approximately one month (i.e., sexual maturity). The numbers of cells projected onto each cluster are given in Supplementary Tables S15 and S17 for humans and mice, respectively. To obtain data for this figure, the minimum projection score for both sets of projections was 0.8. Projections of individual samples are shown in the Supplementary Text.

To gain further insight into the nature of states 0, 0A, and 0B, we applied a two-step projection strategy following the time course of human development. The first step was to project cells from a given sample onto the whole-testis atlas, thereby distinguishing germline from somatic cells. The second step was to project only these putative germ cells onto the SPG atlas. This strategy is conservative but necessary given that the germ cell atlas contains, by definition, only germ cells and that by projecting onto it any given sample (testicular biopsy), non-germ cells would inevitably also be projected. To validate this approach, we confirmed that FACS-sorted undifferentiated spermatogonial populations could be projected onto the map in their expected location. To this end, we obtained three sets of purified spermatogonia from a previous study [24], sorted as HLA-ABC^negative^, CD49e^negative^, THY1^dim^, *ITGA6*+, and EpCAM^dim^—markers that xenotransplantation studies have shown selectively enrich for human spermatogonia with colonization potential [76]. We found that cells in each dataset projected almost entirely onto states 0 and 0B, as expected given that they express the markers used for enrichment (Supplementary Figure S12). Accordingly, we assessed whether each of the undifferentiated cell states were present throughout the human life cycle by conservative projection of a time course of 62 samples, inclusive of the 34 samples used to construct the atlas, and incorporating foetal (8–23 week), pre-, and peripubertal timepoints (Supplementary Table S15). Indeed, we were particularly interested in the relative contribution of the foetal samples to each undifferentiated cell state, as primordial germ cells (PGCs) exist in a state of mitotic arrest [29,52] until their progressive re-entry into the cell cycle shortly after birth. Notably, we found that the (mitotically arrested) PGCs of the foetal samples projected exclusively onto state 0B (Figure 2A and Supplementary Table S15).

State 0B is typified by the expression of the conserved RNA-binding protein *NANOS2* (Supplementary Figure S7), a male-specific stem cell regulator that suppresses the proliferation and differentiation of mouse SSCs and is essential to their self-renewal [77]. Supporting this function, conditional disruption of *Nanos2* in adult mouse testes has long been known to deplete the SSC pool, evidenced by the suppression of spermatogenesis and the loss of *Gfra1+* SSCs [78] (note that in mice *Nanos2*+ germ cells show high overlap with *Gfra1*+ cells [77] but that in humans *GFRA1*+ is characteristic of the ‘active’ state 0A/1). Nevertheless, while *Nanos2* is detectably expressed in the adult mouse testis, it has its strongest and most consistent peak of expression around embryonic days 13–15, when the germline is becoming established and PGCs are mitotically arrested [52,79] (see also https://tanglab.shinyapps.io/Mouse_Male_Germ_Cells/, accessed 27 June 2023). That human PGCs also have a *NANOS2*+ profile supports the conservation of its prenatal function. During this period, PGCs in female and male gonads either, respectively, enter the meiotic programme or mitotic quiescence [80,81]. In males, the role of *Nanos2* is to suppress entry into meiosis by targeting RNAs involved in meiotic initiation, leading to their degradation [82,83,84,85].

A more recent study that performed *Nanos2* knockout on embryonic male germ cells found that *Nanos2* expression also induces mitotic arrest by repressing the activity of mTORC1 [86], a key component of the mTOR signalling pathway that coordinates cell growth and metabolism in response to both environmental and intracellular stressors, including hypoxia and DNA damage [87]. More specifically, *Nanos2* sequesters mTOR in cytoplasmic mRNPs (messenger ribonucleoproteins), a mechanism that allows for considerable flexibility as it permits transient, rapidly reversible repression of cell growth [88]. Conceivably, this mechanism could ‘pause’ cellular mutation independent of, or in addition to, classical (cyclin-based) mechanisms of cell cycle control. Also consistent with a ‘pausing’ or ‘suppressive’ role of state 0B, genes differentially upregulated only in state 0B (Supplementary Table S7) include *GAS5* (‘growth arrest-specific transcript 5’, the overexpression of which induces apoptosis and growth arrest in cancer cells) [89] and *MDH1* (which regulates p53-dependent cell-cycle arrest and apoptosis in response to glucose deprivation) [90]. In addition, a pathway impact analysis (see Section 2) comparing the set of genes differentially expressed in state 0B relative to state 0 found that the KEGG ‘RNA degradation’ pathway was significantly activated; by contrast, it was significantly inhibited in the ‘early diff SPG’ to ‘state 0’ comparison (Supplementary Table S12). Characteristics of state 0B beyond *NANOS2* upregulation are discussed further in the Supplementary Text.

A tantalising explanation for why proliferation would be ‘paused’ is to facilitate DNA damage repair prior to differentiation. Consistent with this, GO terms enriched among the set of genes differentially upregulated only in state 0B—which include *ERCC3*, a nucleotide excision repair gene [91], *TMEM39B*, which in zebrafish protects against oxidative DNA damage induced by cold stress [92], *PSTK*, a phosphorylase kinase with a protective role against oxidative stress [93], *RPL3*, involved in multiple DNA repair pathways in response to chemotherapy [94], and *WWOX*, which modulates the double-strand break response [95,96]—include ‘positive regulation of response to DNA damage stimulus’ and ‘positive regulation of apoptotic signalling pathway’ (Supplementary Table S10). Moreover, a pairwise ‘state 0B relative to state 0’ comparison shows enrichment for the GO term ‘positive regulation of DNA repair’ (Supplementary Table S11).

### 3.5. Heterogeneity of Undifferentiated Spermatogonial States across Species and Development

**Figure 3:**
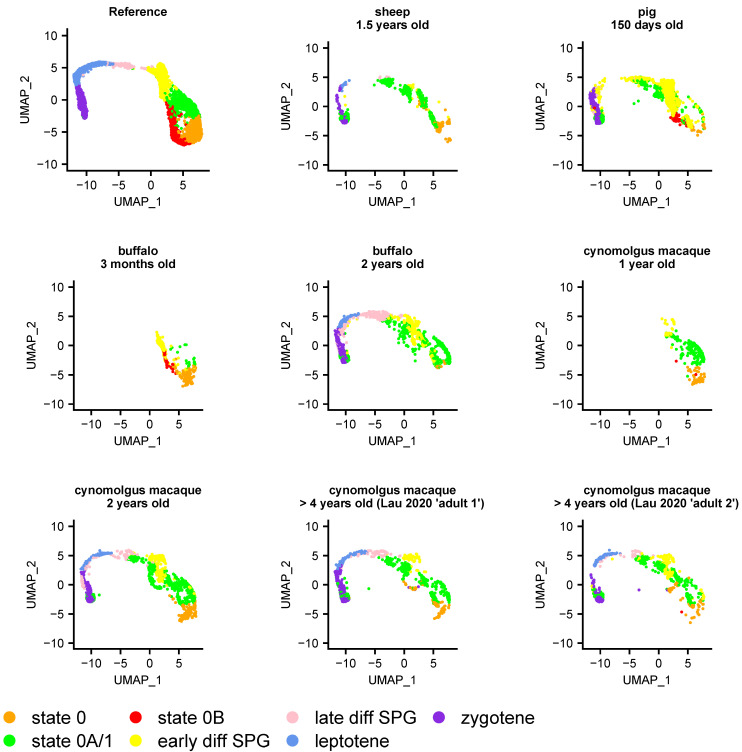
Projection of sheep, pig, buffalo, and cynomologus macaque germ cells onto the SPG atlas, as indicated on the figure. The 3-month-old buffalo and 1-year-old macaque samples are pre-pubertal; all others represent sexually mature individuals (e.g., the two macaques from [30]). These projections show that human state 0 and state 0A-like populations are consistently present in these species, but not necessarily state 0B. Details of the samples included in this analysis are given in Supplementary Table S2. The number of cells projected onto each cluster, for a range of minimum projection scores (0 to 0.8 at 0.2 intervals), are given in Supplementary Table S16, with this figure plotted with no minimum threshold required.

Having established the heterogeneity of undifferentiated spermatogonial states during human development (Figure 2A), we considered to what extent they varied across species. We first projected data from five mammalian testes datasets most closely related to human (rhesus and cynomolgus macaques, plus sheep, pig, and buffalo) to determine whether they had similar undifferentiated spermatogonial states to the human one. In each species, cells equivalent to states 0 and 0A could be identified (Figure 3 and Supplementary Figures S13 and S14).

This is consistent with a shared origin of spermatogenesis in related species and a common transcriptional programme underpinning SSC maturation. Curiously, however, cells projecting onto state 0B belonged primarily to pre-pubertal samples, most notably for a 150-day old pig and a 3-month-old buffalo (Figure 3), but also 15–20-month-old rhesus macaques (Supplementary Figures S13 and S14), with none or a negligible number in sexually mature sheep, buffalo, or macaques. This was surprising, as state 0B in humans is distinguished largely by *NANOS2* expression alone (but see the Supplementary Text). To rule out the possibility that the apparent absence of adult cells with a state 0B signature was an artefact of stochastic capture, we relaxed the Seurat scoring threshold used for the projections (see Section 2), reducing it from a conservative 0.8 (as used in Figure 2A) to 0, thereby allowing the projection of any given cell to its ‘best match’ on the human UMAP. However, even with lenient parameters, state 0B cells were found to be largely absent in these non-human adults (Figure 3). Unsurprisingly they remained so when repeating the analysis with more conservative scoring thresholds (Supplementary Table S16).

**Figure 4:**
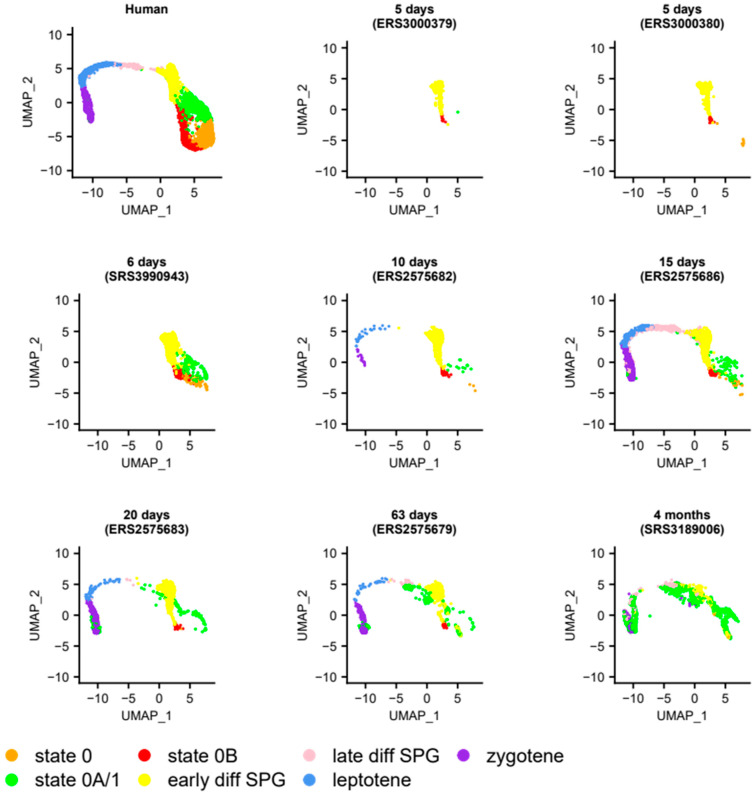
Projection of mouse germ cells onto the SPG atlas, using selected samples representing a time course from early development to adulthood, as indicated on the figure. These projections show that the transcriptomic profile of mouse SSCs varies rapidly across development relative to human ones, with the loss within approximately one month of state 0 and 0B-like populations. Details of the samples included in this analysis are given in Supplementary Table S2, with the relative abundance of each cluster across the full set of 37 samples shown in Figure 2B. The full projection of every sample is given in the Supplementary Text. The number of cells projected onto each cluster, for a range of minimum projection scores (0 to 0.8 at 0.2 intervals), are given in Supplementary Table S17, with this figure plotted with no minimum threshold required.

Together, these results suggest that in these species, state 0B plays a role in early development and that its species-specific retention into adulthood, as well as its relationship to state 0, is of particular importance to steady-state spermatogenesis. To further explore these findings, we considered at what point in development undifferentiated spermatogonial states could be ‘lost’, and why that might be. We were unable to address this with the above species as there were too few samples to reconstruct a time course of development. Accordingly, we projected an extensive time course of 37 unsorted mouse testicular samples (Figure 2B and Figure 4 and Supplementary Table S17), starting from postnatal day (P) five and continuing to adulthood (detailed in Supplementary Table S2). In the two postnatal-day (P)-five murine testis samples, the majority of cells projected not onto the undifferentiated spermatogonial but the ‘early differentiating spermatogonia’ cluster (Figure 4).

**Figure 5:**
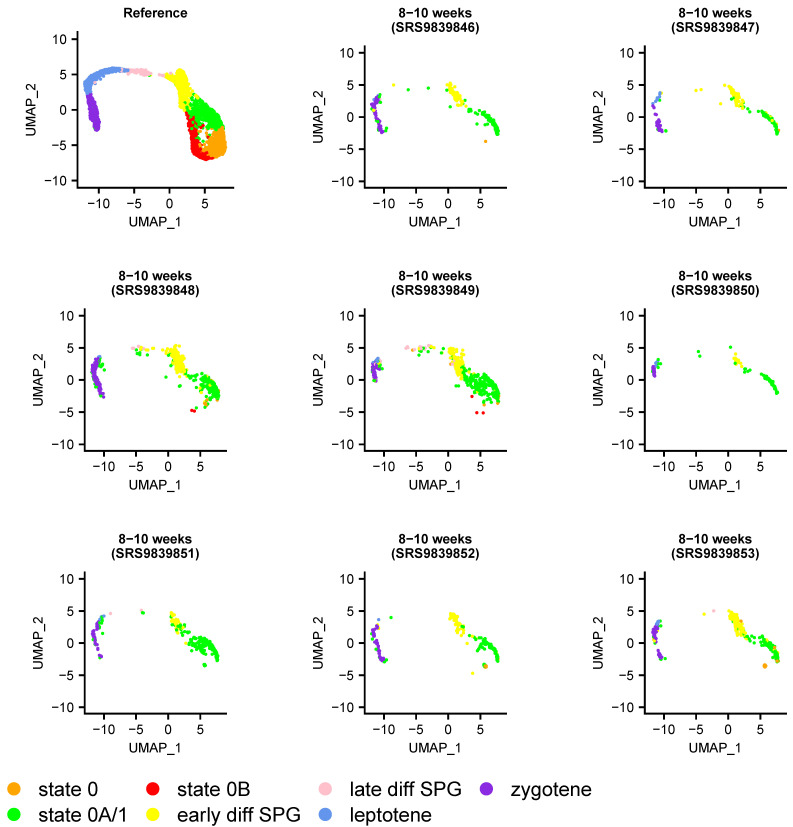
Projection of EpCAM+ selected adult rat germ cells onto the SPG atlas. These projections show that the transcriptomic profile of rat SSCs largely approximates human state 0A. The number of cells projected onto each cluster, for a range of minimum projection scores (0 to 0.8 at 0.2 intervals), are given in Supplementary Table S16, with this figure plotted with no minimum threshold required.

Since in mice, progressive re-entry of PGCs into the cell cycle (after a prolonged period of mitotic arrest) commences around P1–P3 and is completed by P5, this suggests that from a transcriptomic standpoint, P5 germ cells more strongly resemble those inclined to differentiate than undifferentiated spermatogonia. The P5 germ cells are also markedly different to those at P6, in which each of the human-like undifferentiated spermatogonial clusters (states 0, 0A, and 0B) had been established. These observations relate to the first wave of mouse spermatogenesis [97] and are discussed further in the Supplementary Text. From P10 onwards, the meiotic programme was initiated, with a concomitant loss of cells projecting to states 0 and 0B. It appears that for adult mice, essentially the sole transcriptomic programme of undifferentiated spermatogonia resembles human state 0A (Figure 2B). One possible explanation for this observation is that, as state 0A is enriched for OXPHOS metabolism, and because mice, having proportionately fewer undifferentiated spermatogonia than humans [6], must rely on more rounds of energetically expensive transit amplification to produce mature sperm, then in any given mouse testicular biopsy, there may simply be too few SSCs to project onto any other state. Therefore, to further confirm that cells with a human state 0 signature were absent in adult mice, we projected two sorted populations enriched for undifferentiated germ cells (from [98]) onto the human UMAP and obtained the same results (Supplementary Figure S15). Moreover, when projecting additional data from eight adult rat samples, all *EpCAM*+ selected and thereby enriched for germ cells, we found that they also lacked cells with states 0 or 0B signatures (Figure 5 and Supplementary Table S16; EpCAM was detected in 68% of undifferentiated human spermatogonia [see Supplementary Table S7]).

Finally, when projecting murine PGCs from embryonic day 16.5 (from [99]) onto the human UMAP, we noted that both states 0 and 0B (but not 0A) were present (Supplementary Figure S16); the implication is that they differentiate at birth to form the state 0A population.

Overall, these results suggest that, compared to the other mammals considered in this study, the spermatogenic trajectory in rodents follows a different, more developmentally mature, transcriptomic program. More broadly, these results suggest that as with many significant evolutionary steps, spermatogenesis may have been shaped by changes in the sequence of developmental timings, a process known as heterochrony [100].

### 3.6. General Discussion

Although spermatogenesis is often viewed as a highly conserved process, our findings suggest that the origin, developmental timing, and steady-state regulation of the SSC pool differs across species. While not unexpected from an evolutionary perspective [101], the implications of such heterogeneities have not been fully explored in previous single-cell studies, including in refs. [20,21], both of which posit broadly analogous transcriptional states between species across the spermatogonial trajectory, with a common origin in a seemingly homogenous ‘SSC cluster’ or ‘consensus state’. Here we show that the transcriptome of undifferentiated spermatogonia (more so than that of other more mature germ cells) varies greatly between species and across developmental time. In particular, the state 0B signature identifies SSCs that are present during the pubertal development of multiple mammalian species but persisting into adulthood only in humans. This 0B population of undifferentiated spermatogonia has a mitotically poised or ready-to-react transcriptional profile, suggesting its potential role as a reserve population. The relative presence and absence of analogous undifferentiated spermatogonial states between species suggest that SSCs have adopted different strategies to maintain steady-state spermatogenesis. Stem cell behaviour may have been fine-tuned to ensure the preservation of both fertility and genomic integrity over vastly differing life histories and reproductive timeframes, and we hypothesise that the different transcriptomic and transitional states within the undifferentiated spermatogonial population provide a glimpse into the differing evolutionary strategies.

One interpretation of the data is that human SSCs have both ‘slow-cycling’ and ‘fast-cycling’ transcriptomic programmes, shifting seamlessly from the former to the latter, a model not conceptually dissimilar to that of haemopoietic stem cells. Human HSCs comprise three subpopulations [102]: dormant (long-term residency in G_0_), homeostatic/steady-state (occasionally entering the cell cycle and characterised by low metabolic activity), and activated (cycling continuously, and from which a proportion will differentiate). While it is in principle possible to project human HSCs onto the SPG atlas to determine whether dormant/homeostatic/active HSC subpopulations correspond to their equivalents in this system, in practice this is not a viable option. This is because HSC datasets are typically FACS-sorted on the basis of markers not widely expressed in undifferentiated spermatogonia, most notably *CD34* (which was only detected in <5% of SSCs; Supplementary Table S7), leaving too few cells for a meaningful analysis. Nevertheless, it may still be instructive to draw a parallel between HSCs and human SSCs, in which state 0 comprises ‘homeostatic’ SSCs (and possibly also a subset transitioning to or from quiescence) and state 0A ‘activated’ SSCs (which have metabolically shifted to OXPHOS). While we concluded that the state 0 cluster was not as a whole quiescent, we could not rule out the possibility that some cells had a direct relationship to an undefined and hard-to-isolate quiescent state. While we also found no obvious analogue of the ‘dormant’ HSC state in SSCs, the transcriptional programme of state 0B appeared ‘suppressive’, which may be functionally equivalent.

Another stem cell system that may parallel the strategies adopted by SSCs is that of neural stem cells (NSCs). We have shown above that there is little transcriptomic evidence of undifferentiated spermatogonia showing G_0_ quiescence. However, while ‘quiescence’ is conventionally equated with G_0_ (by analogy to a state of nutrient-withdrawal-induced arrest in yeast [103]), this has been challenged by the finding that in *Drosophila*, quiescent neural stem cells arrest heterogeneously, with 75% residing in G_2_ [104]. Similarly, mice have two types of quiescent NSC: ‘dormant’ (which have not previously proliferated) and ‘resting’ (which have proliferated at least once before), analogous to G_0_ and G_2_ quiescence, respectively, with the latter reactivating more quickly [105]. An alternative interpretation of our analysis is that, like NSCs, SSCs also arrest heterogeneously. Consistent with this, a high proportion of cells in state 0 are in G_2_/M (48%; Supplementary Table S13), although also of note is that STK11/LKB1, which is implicated in establishing germline G_2_ quiescence in the ‘dauer’ state of *C. elegans* [106,107], is differentially expressed only in state 0 (Supplementary Table S7). Interestingly, state 0B appears more likely than state 0 to be held at G_2_, as it not only shows a higher proportion of cells in that phase (67%, the highest proportion of G_2_/M cells for all three undifferentiated spermatogonial states; Supplementary Table S13) but has a plausible biological reason to be arrested at that point, namely DNA repair to maintain germline integrity (high-fidelity double-strand break repair can only occur during the S/G_2_ phases because it requires a homologous repair template [105]). Indeed, each of states 0, 0A, and 0B is skewed towards G_2_/M and away from G_1_—but state 0B most of all, with only 5% of its cells in G_1_, in contrast to 9% for state 0A and 12% for state 0 (Supplementary Table S13; see also the Supplementary Text). The relative proportion of G_2_/M to S is also seemingly more skewed for 0B than the other states. The G_2_/M to S ratio for states 0 and 0A approximates parity, whereas for state 0B, it appears skewed by 2:1 towards G_2_/M (Supplementary Table S13). A prolonged G_2_ phase has been associated with regeneration in other systems (including muscle stem cells in zebrafish embryos [108,109], adult stem cells in the intestinal crypts of the naked mole rat, the longest-living rodent [110], and adult stem cells of the regenerative *Hydra* polyp, which alternate between G_2_ and S, with minimal G_1_ [111]), which is of note here given the selective pressure to maintain low mutation rates in SSCs. A conceptual advance offered to SSC biology by NSCs is suggesting that a reserve population (if present) may not be ‘dormant’ or ‘outside the cell cycle’ at all but ‘resting’, ‘waiting’, or ‘paused’; that is, not compromising the ability to be ready to react (parallels between NSCs and SSCs are discussed further in the Supplementary Text).

With the transcriptomic programmes of undifferentiated spermatogonia broadly outlined, our most notable finding is that in a cross-species comparison, states analogous to human states 0 and 0B were absent from adult rats and mice. We should recall at this point that the expression profiles of states 0 and 0B are slightly more correlated with each other than either are to state 0A (Supplementary Figure S10). In this respect and given states 0 and 0B are ‘not activated’ (i.e., not showing OXPHOS metabolism), they may have a particularly entwined set of functions. As a programme of coordinated expression but unclear function, we conjectured state 0B is ‘suppressive’ both because it is (in part) transcriptionally directed towards state 0 (Supplementary Figure S11) and because during early development the function of its main diagnostic marker, *NANOS2*, is to induce mitotic arrest and degrade RNAs involved in meiotic entry [86]. Consistent with a role in slowing down cellular maturation, candidate target genes for NANOS2 include *Taf7l*, *Dazl*, *Sycp3*, and *Prdm9* [83], the former differentially expressed immediately downstream of state 0B, in state 0A/1, the middle two differentially expressed in the ‘late diff SPG’, ‘leptotene’, and ‘zygotene’ clusters, and the latter, a key regulator of meiotic double-strand breaks [112], differentially expressed only in ‘leptotene’ (Supplementary Table S7). Nevertheless, the full suite of NANOS2 targets and interacting partners remains elusive, and its precise function in adults is a current gap in our knowledge. For instance, a previous study in mice found that Nanos2 requires a germ-cell-specific RNA-binding protein, Dnd1, to associate with its target RNAs [113]; curiously, we found no evidence of its orthologue in our data (Supplementary Table S7).

However, assuming state 0B is a dedicated ‘suppressive program’, why would adult rodents not require this? Although the basic elements of spermatogenesis are conserved between rodents and humans [6], the degree of transcriptomic similarity between undifferentiated primate and rodent spermatogonia is relatively low [5]. We have noted above that in mice, undifferentiated spermatogonia expressing *Nanos2* are most likely to also express *Gfra1* [77] and that accordingly their closest human counterpart resembles state 0A, not 0B. These *Gfra1*+ *Nanos2*+ cells self-renew while producing a differentiation-primed *Ngn3*+ *Miwi2*+ *Rarγ*+ population that differentiates without self-renewal (reviewed in [114])—cells which should in principle be analogous to the human ‘early diff SPG’ cluster if the species’ trajectories were similar. However, based on our data, this does not appear to be the case: in human spermatogonia, *PIWIL4* (*Miwi2*) is differentially expressed in state 0 (i.e., downstream to where it is expressed in mice), *RARG* (*Rarγ*) shows no evidence of differential expression in any cluster of the SPG atlas, and *NEUROG3* (*Ngn3*) is not detectable at all (Supplementary Table S7). Notable differences between the primate and rodent germline have been reported before. For example, in humans, SOX17 is a critical regulator of PGC fate [115] but plays no role in the specification of the mouse germline [116]. Mice also maintain a smaller stem cell pool than humans and compensate with a greater number of transit amplification divisions, ultimately producing around ten times more sperm per gram of testis parenchyma per day [6]. Interestingly, these species-specific features may relate to their known differences in age at sexual maturity and lifespan. Using estimates of the average age of male sexual maturity from the AnAge database [117], it is apparent that mice are able to produce large volumes of sperm comparatively rapidly, entering puberty practically at birth and accomplishing in two months what in humans takes around 14 years (Supplementary Table S18). As such, it is possible that relatively longer-lived species retain into adulthood the otherwise foetal state 0B, so as to reduce mutagenic load over the long term while ensuring the regular production of gametes across their longer reproductive lives. Consistent with this, the germline mutation rate of humans is an order of magnitude lower than mice (the ‘modeled rate per year’ from ref. [1] is 3.52 × 10^−9^ for mice and 4.18 × 10^−10^ for human). Overall, we suggest that shorter-lived species have adopted a different evolutionary strategy for producing sperm—one where undifferentiated adult spermatogonia do not employ the state 0B programme and where there is little selective benefit of ‘waiting’.

## Data Availability

All raw sequencing data were sourced from openly available databases using the accession numbers given in Supplementary Table S1. An interactive version of the SPG atlas, made using ShinyCell [118], is available at https://trainingidn.shinyapps.io/hu_spermatogonial_atlas_shinyApp. All scripts used to perform the analyses and create both figures and tables are available at www.github.com/sjbush/spg_atlas.

## Supplementary Materials

The following supporting information can be downloaded at: https://www.mdpi.com/article/10.3390/cells13090742/s1, Text S1: supplementary text; Table S1: source of testes scRNA-seq datasets; Table S2: scRNA-seq sample metadata; Table S3: number of cells, genes, and average genes per cell, per sample; Table S4: cell and spermatogonial stem cell state biomarkers; Table S5: adult human testis expression atlas; Table S6: adult human SPG expression atlas (Seurat clustering resolution 0.3); Table S7: adult human SPG expression atlas (Seurat clustering resolution 1.1); Table S8: adult human SPG expression atlas (Seurat clustering resolution 1.2); Table S9: enriched biological process GO terms for genes differentially expressed in the adult testis atlas; Table S10: enriched biological process GO terms for genes differentially expressed in the SPG atlas (in an all-against-all analysis); Table S11: enriched biological process GO terms for genes differentially expressed in the SPG atlas (in an X-against-Y analysis); Table S12: enriched KEGG pathways for genes differentially expressed in the SPG atlas (in an X-against-Y analysis); Table S13: proportion of cells in each SPG atlas cluster in each cell cycle phase; Table S14: expression of quiescence-associated genes in undifferentiated spermatogonial states 0, 0A and 0B; Table S15: proportion of cells from 62 human samples projecting to each of seven SPG atlas clusters; Table S16: proportion of cells from 1 sheep, 1 pig, 2 buffalo, 4 cynomolgus macaque, 11 rhesus macaque, and 8 rat samples projecting to each of seven SPG atlas clusters; Table S17: proportion of cells from 37 mouse samples projecting to each of seven SPG atlas clusters; Table S18: average age of sexual maturity and maximum lifespan; Table S19: proportion of cluster-enriched genes, from Huang, et al. (2023), detectable in the SPG atlas; Table S20: proportion of core genes, from Murat, et al. (2023), detectable in the SPG atlas; Table S21: gene expression in mouse germ cells from postnatal days 5 and 6; Table S22: cell cycle phase predictions for the cells comprising the SPG atlas; Figure S1: no overt batch effects in the adult testis atlas on the basis of study of origin (A), donor age (B), or sample accession (C). Figure S2: no overtly disproportionate contribution made by individual samples to the adult testis atlas, on the basis of number of genes per cell (A), number of UMIs per cell (B), and percentage of mitochondrial genes per cell (C). Boxes represent the interquartile range of each variable, with midlines representing the median. Upper and lower whiskers extend, respectively, to the largest and smallest values no further than 1.5x the interquartile range. Data beyond the ends of each whisker are outliers and plotted individually. Figure S3: No overt batch effects in the adult SPG atlas on the basis of study of origin (A), donor age (B), or sample accession (C). Figure S4: No overtly disproportionate contribution made by individual samples to the adult SPG atlas, on the basis of number of genes per cell (A), number of UMIs per cell (B), and percentage of mitochondrial genes per cell (C). Boxes represent the interquartile range of each variable, with midlines representing the median. Upper and lower whiskers extend, respectively, to the largest and smallest values no further than 1.5x the interquartile range. Data beyond the ends of each whisker are outliers and plotted individually. Figure S5: proportional contribution of UMIs made by each (A) sample and (B) study to each cluster in the adult SPG atlas. Figure S6: reciprocal projection of (A) the ‘undiff SPG’ and ‘diff SPG’ clusters of the whole-testis atlas onto the SPG atlas, and (B) the SPG atlas onto the whole-testis atlas, to confirm that the two atlases shared the same cell populations. Figure S7: relative expression, across the SPG atlas, of key genes spanning the temporal order of gametogenesis. This figure shows the relative expression, across the SPG atlas, of 16 genes using a yellow (minimum) to red (maximum) colour gradient. Note that each plot has been individually scaled to the maximum expression value of that gene and so the colour scales are not comparable across plots (and so are not shown). For illustrative purposes, it suffices to note that the maximum expression of each gene is confined to a particular cell type, and is widely considered a discriminative biomarker of it (as detailed in Supplementary Table S4). Figure S8: Clustree [55] plot showing the effect of different resolutions on the cluster composition of the SPG atlas. Clustering was performed using Seurat with resolution varied from 0 to 2 at 0.1 intervals. Seurat labels clusters according to their size, with “cluster 0” the largest. The set of nodes on each row represent the clusters formed at each resolution (i.e. at resolution 0, there is one node, and at resolution 0.1, three nodes). Arrows between nodes indicate their relationship to clusters formed at the next level of resolution. For example, as resolution increases from 0.2 to 0.3, the “cluster 0” node is subset into two nodes, “cluster 1” and “cluster 2”, indicating that the cluster has been broken up. The relative size, colour and thickness of each arrow indicate the proportion of cells contributed to each cluster. Figure S9: single-cell expression atlas of spermatogonia and their progenies, at Seurat clustering resolutions (A) 0.3, (B) 1.1, and (C) 1.2, these partitioning the undifferentiated spermatogonial compartment into two, three, and four subsets, respectively. Note that panel B is identical to Figure 1B. Figure S10: relative expression of genes in each of the three undifferentiated spermatogonial clusters (states 0, 0A/1, and 0B), using the SPG atlas clustered at resolution 1.1. The figure plots, per gene, the mean expression across all cells in the state 0 cluster relative to all cells in the state 0A/1 cluster (red circles), and to all cells in the state 0B cluster (blue triangles), demonstrating a substantial overlap between the three. There are significantly high Spearman’s correlations between all three comparisons (*rho* for state 0 vs. state 0A/1 = 0.934; for state 0 vs. state 0B = 0.955; for state 0A/1 vs. state 0B = 0.939; *p* < 2.2 × 10^−16^ in each case). Expression levels are quantified by Seurat and have arbitrary units. Raw data for this figure is available in Supplementary Table S7. Figure S11: visualisation of the results of an scVelo RNA velocity analysis on the SPG atlas UMAP. The streamlines indicate developmental trajectories, with the direction and thickness of each representing the orientation of the trajectory and the magnitude of its velocity, respectively. Strong and unidirectional streamlines are most notable in the ‘early diff SPG’ cluster, proceeding leftwards to the onset of meiosis. There are similarly strong directional streamlines proceeding through the early stages of prophase (where the trajectory is terminated) although nevertheless a number of backward-pointing arrows between stages, interpreted here to indicate the activity of the meiotic checkpoint network [41] that regulates progression. Moving to the right of the figure, we can see that the directionality of the cells is less certain. Between the undifferentiated spermatogonial ring and the ‘early diff SPG’ cluster, there are multiple clear streamlines pointing both up (towards differentiation) and down, consistent with previous suggestions that prior to differentiation, undifferentiated spermatogonia are not irreversibly committed to their fate and may instead show more plastic behaviour [10]. Streamlines within the ring are discussed in the main text. Figure S12: verification of the two-step projection strategy. (A) Projection of three FACS-sorted steady-state spermatogonial samples (from [24]) onto the adult testis atlas, so as to distinguish germline from somatic cells. (B) Projection of these putative germ cells onto the SPG atlas. Figure S13: projection of rhesus macaque germ cells onto the SPG atlas, part 1 of 2. Details of the samples included in this analysis are given in Supplementary Table S2. The number of cells projected onto each cluster, for a range of minimum projection scores (0 to 0.8 at 0.2 intervals), are given in Supplementary Table S16, with this figure plotted with no minimum threshold required. Figure S14: projection of rhesus macaque germ cells onto the SPG atlas, part 2 of 2. Details of the samples included in this analysis are given in Supplementary Table S2. The number of cells projected onto each cluster, for a range of minimum projection scores (0 to 0.8 at 0.2 intervals), are given in Supplementary Table S16, with this figure plotted with no minimum threshold required. Figure S15: confirmation that human states 0 and 0B-like populations are not obviously apparent in adult mice. (A) Projection of two sorted populations of undifferentiated mouse germ cells (from [98]) onto the adult testis atlas, so as to distinguish germline from somatic cells. (B) Projection of these germ cells onto the SPG atlas. These two samples are biological replicates from 8–10 week old mice and represent cells isolated from the testes on the basis of *Plzf* (ZBTB16) gene expression, and then stained for CD9 and c-Kit, markers of stem and differentiating spermatogonia, respectively. As both CD9 and ZBTB16 are detected in a comparably high number of cells in each of human states 0, 0A and 0B (Supplementary Table S7; note that ZBTB16 is differentially expressed in state 0, too), we considered it reasonable to perform a mouse-to-human projection. Note that CD9 is also expressed by somatic cells and so a small proportion of each sample are excluded by the two-step projection strategy (because some cells project to the myoid/Leydig cluster rather than the germline). Figure S16: presence of human states 0 and 0B-like populations in embryonic mice. (A) Projection of three populations of embryonic day 16.5 mouse PGCs (from [99]) onto the adult testis atlas, so as to distinguish germline from somatic cells. (B) Projection of these germ cells onto the SPG atlas.

## References

[1] Bergeron L.A., Besenbacher S., Zheng J., Li P., Bertelsen M.F., Quintard B., Hoffman J.I., Li Z., Leger J.S., Shao C. (2023). Evolution of the germline mutation rate across vertebrates. Nature.

[2] Seplyarskiy V.B., Sunyaev S. (2021). The origin of human mutation in light of genomic data. Nat. Rev. Genet..

[3] Bush S.J., Goriely A. (2023). Fine-tuning germline mutation rates across evolution. Trends Genet..

[4] Nakagawa T., Jörg D.J., Watanabe H., Mizuno S., Han S., Ikeda T., Omatsu Y., Nishimura K., Fujita M., Takahashi S. (2021). A multistate stem cell dynamics maintains homeostasis in mouse spermatogenesis. Cell Rep..

[5] Singh A., Hermann B.P. (2023). Conserved Transcriptome Features Define Prepubertal Primate Spermatogonial Stem Cells as A_dark_ Spermatogonia and Identify Unique Regulators. Int. J. Mol. Sci..

[6] Fayomi A.P., Orwig K.E. (2018). Spermatogonial stem cells and spermatogenesis in mice, monkeys and men. Stem Cell Res..

[7] Clermont Y. (1963). The cycle of the seminiferous epithelium in man. Am. J. Anat..

[8] Heller C.G., Clermont Y. (1963). Spermatogenesis in man: An estimate of its duration. Science.

[9] Clermont Y. (1966). Spermatogenesis in man. A study of the spermatogonial population. Fertil. Steril..

[10] Guo J., Grow E.J., Mlcochova H., Maher G.J., Lindskog C., Nie X., Guo Y., Takei Y., Yun J., Cai L. (2018). The adult human testis transcriptional cell atlas. Cell Res..

[11] Guo J., Grow E.J., Yi C., Mlcochova H., Maher G.J., Lindskog C., Murphy P.J., Wike C.L., Carrell D.T., Goriely A. (2017). Chromatin and Single-Cell RNA-Seq Profiling Reveal Dynamic Signaling and Metabolic Transitions during Human Spermatogonial Stem Cell Development. Cell Stem Cell.

[12] von Kopylow K., Spiess A.-N. (2017). Human spermatogonial markers. Stem Cell Res..

[13] Di Persio S., Saracino R., Fera S., Muciaccia B., Esposito V., Boitani C., Berloco B.P., Nudo F., Spadetta G., Stefanini M. (2017). Spermatogonial kinetics in humans. Development.

[14] Di Persio S., Neuhaus N. (2023). Human spermatogonial stem cells and their niche in male (in)fertility: Novel concepts from single-cell RNA-sequencing. Hum. Reprod..

[15] Di Persio S., Tekath T., Siebert-Kuss L.M., Cremers J.F., Wistuba J., Li X., Zu Hörste G.M., Drexler H.C., Wyrwoll M.J., Tüttelmann F. (2021). Single-cell RNA-seq unravels alterations of the human spermatogonial stem cell compartment in patients with impaired spermatogenesis. Cell Rep. Med..

[16] Sohni A., Tan K., Song H.-W., Burow D., de Rooij D.G., Laurent L., Hsieh T.-C., Rabah R., Hammoud S.S., Vicini E. (2019). The Neonatal and Adult Human Testis Defined at the Single-Cell Level. Cell Rep..

[17] Hara K., Nakagawa T., Enomoto H., Suzuki M., Yamamoto M., Simons B.D., Yoshida S. (2014). Mouse spermatogenic stem cells continually interconvert between equipotent singly isolated and syncytial states. Cell Stem Cell.

[18] Goriely A., Wilkie A.O. (2012). Paternal Age Effect mutations and selfish spermatogonial selection: Causes and consequences for human disease. Am. J. Hum. Genet..

[19] Wood K.A., Goriely A. (2022). The impact of paternal age on new mutations and disease in the next generation. Fertil. Steril..

[20] Shami A.N., Zheng X., Munyoki S.K., Ma Q., Manske G.L., Green C.D., Sukhwani M., Orwig K.E., Li J.Z., Hammoud S.S. (2020). Single-Cell RNA Sequencing of Human, Macaque, and Mouse Testes Uncovers Conserved and Divergent Features of Mammalian Spermatogenesis. Dev. Cell.

[21] Murat F., Mbengue N., Winge S.B., Trefzer T., Leushkin E., Sepp M., Cardoso-Moreira M., Schmidt J., Schneider C., Mößinger K. (2022). The molecular evolution of spermatogenesis across mammals. Nature.

[22] Chen S., An G., Wang H., Wu X., Ping P., Hu L., Chen Y., Fan J., Cheng C.Y., Sun F. (2022). Human obstructive (postvasectomy) and nonobstructive azoospermia—Insights from scRNA-Seq and transcriptome analysis. Genes Dis..

[23] Zhao L., Yao C., Xing X., Jing T., Li P., Zhu Z., Yang C., Zhai J., Tian R., Chen H. (2020). Single-cell analysis of developing and azoospermia human testicles reveals central role of Sertoli cells. Nat. Commun..

[24] Hermann B.P., Cheng K., Singh A., Roa-De La Cruz L., Mutoji K.N., Chen I.-C., Gildersleeve H., Lehle J.D., Mayo M., Westernströer B. (2018). The Mammalian Spermatogenesis Single-Cell Transcriptome, from Spermatogonial Stem Cells to Spermatids. Cell Rep..

[25] Guo J., Nie X., Giebler M., Mlcochova H., Wang Y., Grow E.J., Kim R., Tharmalingam M., Matilionyte G., DonorConnect (2020). The Dynamic Transcriptional Cell Atlas of Testis Development during Human Puberty. Cell Stem Cell.

[26] Nie X., Munyoki S.K., Sukhwani M., Schmid N., Missel A., Emery B.R., Connect D., Stukenborg J.-B., Mayerhofer A., Orwig K.E. (2022). Single-cell analysis of human testis aging and correlation with elevated body mass index. Dev. Cell.

[27] Guo J., Sosa E., Chitiashvili T., Nie X., Rojas E.J., Oliver E., Plath K., Hotaling J.M., Stukenborg J.-B., Clark A.T. (2021). Single-cell analysis of the developing human testis reveals somatic niche cell specification and fetal germline stem cell establishment. Cell Stem Cell.

[28] Voigt A.L., Dardari R., Su L., Lara N.L.M., Sinha S., Jaffer A., Munyoki S.K., Alpaugh W., Dufour A., Biernaskie J. (2022). Metabolic transitions define spermatogonial stem cell maturation. Hum. Reprod..

[29] Wang R., Liu X., Li L., Yang M., Yong J., Zhai F., Wen L., Yan L., Qiao J., Tang F. (2022). Dissecting Human Gonadal Cell Lineage Specification and Sex Determination Using a Single-cell RNA-seq Approach. Genom. Proteom. Bioinform..

[30] Lau X., Munusamy P., Ng M.J., Sangrithi M. (2020). Single-Cell RNA Sequencing of the Cynomolgus Macaque Testis Reveals Conserved Transcriptional Profiles during Mammalian Spermatogenesis. Dev. Cell.

[31] Ernst C., Eling N., Martinez-Jimenez C.P., Marioni J.C., Odom D.T. (2019). Staged developmental mapping and X chromo-some transcriptional dynamics during mouse spermatogenesis. Nat. Commun..

[32] Grive K.J., Hu Y., Shu E., Grimson A., Elemento O., Grenier J.K., Cohen P.E. (2019). Dynamic transcriptome profiles within spermatogonial and spermatocyte populations during postnatal testis maturation revealed by single-cell sequencing. PLoS Genet..

[33] Green C.D., Ma Q., Manske G.L., Shami A.N., Zheng X., Marini S., Moritz L., Sultan C., Gurczynski S.J., Moore B.B. (2018). A Comprehensive Roadmap of Murine Spermatogenesis Defined by Single-Cell RNA-Seq. Dev. Cell.

[34] Jung M., Wells D., Rusch J., Ahmad S., Marchini J., Myers S.R., Conrad D.F. (2019). Unified single-cell analysis of testis gene regulation and pathology in five mouse strains. eLife.

[35] Whelan E.C., Yang F., Avarbock M.R., Sullivan M.C., Beiting D.P., Brinster R.L. (2022). Reestablishment of spermatogenesis after more than 20 years of cryopreservation of rat spermatogonial stem cells reveals an important impact in differentiation capacity. PLoS Biol..

[36] Zhang L., Li F., Lei P., Guo M., Liu R., Wang L., Yu T., Lv Y., Zhang T., Zeng W. (2021). Single-cell RNA-sequencing reveals the dynamic process and novel markers in porcine spermatogenesis. J. Anim. Sci. Biotechnol..

[37] Yang H., Ma J., Wan Z., Wang Q., Wang Z., Zhao J., Wang F., Zhang Y. (2020). Characterization of sheep spermatogenesis through single-cell RNA sequencing. FASEB J..

[38] Huang L., Zhang J., Zhang P., Huang X., Yang W., Liu R., Sun Q., Lu Y., Zhang M., Fu Q. (2023). Single-cell RNA sequencing uncovers dynamic roadmap and cell-cell communication during buffalo spermatogenesis. iScience.

[39] Neuhaus N., Yoon J., Terwort N., Kliesch S., Seggewiss J., Huge A., Voss R., Schlatt S., Grindberg R., Schöler H. (2017). Single-cell gene expression analysis reveals diversity among human spermatogonia. Mol. Hum. Reprod..

[40] Li L., Dong J., Yan L., Yong J., Liu X., Hu Y., Fan X., Wu X., Guo H., Wang X. (2017). Single-Cell RNA-Seq Analysis Maps Development of Human Germline Cells and Gonadal Niche Interactions. Cell Stem Cell.

[41] Wang M., Liu X., Chang G., Chen Y., An G., Yan L., Gao S., Xu Y., Cui Y., Dong J. (2018). Single-Cell RNA Sequencing Analysis Reveals Sequential Cell Fate Transition during Human Spermatogenesis. Cell Stem Cell.

[42] Picelli S., Bjorklund A.K., Faridani O.R., Sagasser S., Winberg G., Sandberg R. (2013). Smart-seq2 for sensitive full-length transcriptome profiling in single cells. Nat. Methods.

[43] Tan K., Song H.-W., Thompson M., Munyoki S., Sukhwani M., Hsieh T.-C., Orwig K.E., Wilkinson M.F. (2020). Transcriptome profiling reveals signaling conditions dictating human spermatogonia fate in vitro. Proc. Natl. Acad. Sci. USA.

[44] Laurentino S., Heckmann L., Di Persio S., Li X., Meyer zu Hörste G., Wistuba J., Cremers J.F., Gromoll J., Kliesch S., Schlatt S. (2019). High-resolution analysis of germ cells from men with sex chromosomal aneuploidies reveals normal transcriptome but impaired imprinting. Clin. Epigenet..

[45] Melsted P., Booeshaghi A.S., Liu L., Gao F., Lu L., Min K.H., Beltrame E.d.V., Hjörleifsson K.E., Gehring J., Pachter L. (2021). Modular, efficient and constant-memory single-cell RNA-seq preprocessing. Nat. Biotechnol..

[46] Kinsella R.J., Kähäri A., Haider S., Zamora J., Proctor G., Spudich G., Almeida-King J., Staines D., Derwent P., Kerhornou A. (2011). Ensembl BioMarts: A hub for data retrieval across taxonomic space. Database.

[47] Macosko E.Z., Basu A., Satija R., Nemesh J., Shekhar K., Goldman M., Tirosh I., Bialas A.R., Kamitaki N., Martersteck E.M. (2015). Highly Parallel Genome-wide Expression Profiling of Individual Cells Using Nanoliter Droplets. Cell.

[48] Hao Y., Hao S., Andersen-Nissen E., Mauck W.M., Zheng S., Butler A., Lee M.J., Wilk A.J., Darby C., Zager M. (2021). Integrated analysis of multimodal single-cell data. Cell.

[49] Hafemeister C., Satija R. (2019). Normalization and variance stabilization of single-cell RNA-seq data using regularized negative binomial regression. Genome Biol..

[50] Ahlmann-Eltze C., Huber W. (2020). glmGamPoi: Fitting Gamma-Poisson generalized linear models on single cell count data. Bioinformatics.

[51] Dominguez D., Tsai Y.-H., Gomez N., Jha D.K., Davis I., Wang Z. (2016). A high-resolution transcriptome map of cell cycle reveals novel connections between periodic genes and cancer. Cell Res..

[52] Zhao J., Lu P., Wan C., Huang Y., Cui M., Yang X., Hu Y., Zheng Y., Dong J., Wang M. (2021). Cell-fate transition and determination analysis of mouse male germ cells throughout development. Nat. Commun..

[53] McInnes L., Healy J., Melville J. (2018). Umap: Uniform manifold approximation and projection for dimension reduction. arXiv.

[54] Waltman L., van Eck N.J. (2013). A smart local moving algorithm for large-scale modularity-based community detection. Eur. Phys. J. B.

[55] Zappia L., Oshlack A. (2018). Clustering trees: A visualization for evaluating clusterings at multiple resolutions. GigaScience.

[56] Bergen V., Lange M., Peidli S., Wolf F.A., Theis F.J. (2020). Generalizing RNA velocity to transient cell states through dynamical modeling. Nat. Biotechnol..

[57] Alexa A., Rahnenführer J., Lengauer T. (2006). Improved scoring of functional groups from gene expression data by decorrelating GO graph structure. Bioinformatics.

[58] Tarca A.L., Draghici S., Khatri P., Hassan S.S., Mittal P., Kim J.-S., Kim C.J., Kusanovic J.P., Romero R. (2008). A novel signaling pathway impact analysis. Bioinformatics.

[59] Kanehisa M., Sato Y., Kawashima M., Furumichi M., Tanabe M. (2016). KEGG as a reference resource for gene and protein annotation. Nucleic Acids Res..

[60] Mitrea C., Taghavi Z., Bokanizad B., Hanoudi S., Tagett R., Donato M., Voichiţa C., Drăghici S. (2013). Methods and approaches in the topology-based analysis of biological pathways. Front. Physiol..

[61] Suzuki S., Diaz V.D., Hermann B.P. (2019). What has single-cell RNA-seq taught us about mammalian spermatogenesis?. Biol. Reprod..

[62] Patterson-Cross R.B., Levine A.J., Menon V. (2021). Selecting single cell clustering parameter values using subsampling-based robustness metrics. BMC Bioinform..

[63] Carneiro F.R., Lepelley A., Seeley J.J., Hayden M.S., Ghosh S. (2018). An Essential Role for ECSIT in Mitochondrial Complex I Assembly and Mitophagy in Macrophages. Cell Rep..

[64] Kathagen-Buhmann A., Schulte A., Weller J., Holz M., Herold-Mende C., Glass R., Lamszus K. (2016). Glycolysis and the pentose phosphate pathway are differentially associated with the dichotomous regulation of glioblastoma cell migration versus proliferation. Neuro-Oncology.

[65] van Velthoven C.T.J., Rando T.A. (2019). Stem Cell Quiescence: Dynamism, Restraint, and Cellular Idling. Cell Stem Cell.

[66] Llorens-Bobadilla E., Zhao S., Baser A., Saiz-Castro G., Zwadlo K., Martin-Villalba A. (2015). Single-Cell Transcriptomics Reveals a Population of Dormant Neural Stem Cells that Become Acti-vated upon Brain Injury. Cell Stem Cell.

[67] Simsek T., Kocabas F., Zheng J., DeBerardinis R.J., Mahmoud A.I., Olson E.N., Schneider J.W., Zhang C.C., Sadek H.A. (2010). The Distinct Metabolic Profile of Hematopoietic Stem Cells Reflects Their Location in a Hypoxic Niche. Cell Stem Cell.

[68] Cheung T.H., Rando T.A. (2013). Molecular regulation of stem cell quiescence. Nat. Rev. Mol. Cell Biol..

[69] Li J. (2011). Quiescence regulators for hematopoietic stem cell. Exp. Hematol..

[70] Nakamura-Ishizu A., Takizawa H., Suda T. (2014). The analysis, roles and regulation of quiescence in hematopoietic stem cells. Development.

[71] Zhang Y.W., Mess J., Aizarani N., Mishra P., Johnson C., Romero-Mulero M.C., Rettkowski J., Schönberger K., Obier N., Jäcklein K. (2022). Hyaluronic acid–*GPRC5C* signalling promotes dormancy in haematopoietic stem cells. Nature.

[72] Liao H.-F., Chen W.S.C., Chen Y.-H., Kao T.-H., Tseng Y.-T., Lee C.-Y., Chiu Y.-C., Lee P.-L., Lin Q.-J., Ching Y.-H. (2014). DNMT3L promotes quiescence in postnatal spermatogonial progenitor cells. Development.

[73] Chong S.Y., Cutler S., Lin J.-J., Tsai C.-H., Tsai H.-K., Biggins S., Tsukiyama T., Lo Y.-C., Kao C.-F. (2020). H3K4 methylation at active genes mitigates transcription-replication conflicts during replication stress. Nat. Commun..

[74] Wang Z., Jin C., Li P., Li Y., Tang J., Yu Z., Jiao T., Ou J., Wang H., Zou D. (2023). Identification of quiescent FOXC2(+) spermatogonial stem cells in adult mammals. eLife.

[75] Caldeira-Brant A.L., Martinelli L.M., Marques M.M., Reis A.B., Martello R., Almeida F.R.C.L., Chiarini-Garcia H. (2020). A subpopulation of human Adark spermatogonia behaves as the reserve stem cell. Reproduction.

[76] Dovey S.L., Valli H., Hermann B.P., Sukhwani M., Donohue J., Castro C.A., Chu T., Sanfilippo J.S., Orwig K.E. (2013). Eliminating malignant contamination from therapeutic human spermatogonial stem cells. J. Clin. Investig..

[77] Sada A., Hasegawa K., Pin P.H., Saga Y. (2012). *NANOS2* acts downstream of glial cell line-derived neurotrophic factor signal-ing to suppress differentiation of spermatogonial stem cells. Stem Cells.

[78] Sada A., Suzuki A., Suzuki H., Saga Y. (2009). The RNA-binding protein *NANOS2* is required to maintain murine spermatogonial stem cells. Science.

[79] Pandey V., Tripathi A., Dubey P.K. (2019). Expression and intracellular localization of *NANOS2*-homologue protein in primordial germ cells and spermatogonial stem cells. Zygote.

[80] Bowles J., Knight D., Smith C., Wilhelm D., Richman J., Mamiya S., Yashiro K., Chawengsaksophak K., Wilson M.J., Rossant J. (2006). Retinoid Signaling Determines Germ Cell Fate in Mice. Science.

[81] Koubova J., Menke D.B., Zhou Q., Capel B., Griswold M.D., Page D.C. (2006). Retinoic acid regulates sex-specific timing of meiotic initiation in mice. Proc. Natl. Acad. Sci. USA.

[82] Kato Y., Katsuki T., Kokubo H., Masuda A., Saga Y. (2016). Dazl is a target RNA suppressed by mammalian *NANOS2* in sexually differentiating male germ cells. Nat. Commun..

[83] Suzuki A., Igarashi K., Aisaki K.-I., Kanno J., Saga Y. (2010). *NANOS2* interacts with the CCR4-NOT deadenylation complex and leads to suppression of specific RNAs. Proc. Natl. Acad. Sci. USA.

[84] Suzuki A., Saba R., Miyoshi K., Morita Y., Saga Y. (2012). Interaction between *NANOS2* and the CCR4-NOT deadenylation complex is essential for male germ cell development in mouse. PLoS ONE.

[85] Suzuki A., Niimi Y., Saga Y. (2014). Interaction of *NANOS2* and *NANOS3* with different components of the CNOT complex may contribute to the functional differences in mouse male germ cells. Biol. Open.

[86] Shimada R., Koike H., Hirano T., Kato Y., Saga Y. (2021). *NANOS2* suppresses the cell cycle by repressing mTORC1 activators in embryonic male germ cells. iScience.

[87] Saxton R.A., Sabatini D.M. (2017). mTOR Signaling in Growth, Metabolism, and Disease. Cell.

[88] Zhou Z., Shirakawa T., Ohbo K., Sada A., Wu Q., Hasegawa K., Saba R., Saga Y. (2015). RNA Binding Protein Nanos2 Organizes Post-transcriptional Buffering System to Retain Primitive State of Mouse Spermatogonial Stem Cells. Dev. Cell.

[89] Xu Y., Lu J., Lou N., Lu W., Xu J., Jiang H., Ye G. (2022). Long noncoding RNA GAS5 inhibits proliferation and metastasis in papillary thyroid carcinoma through the IFN/STAT1 signaling pathway. Pathol. Res. Pr..

[90] Lee S.M., Kim J.H., Cho E.J., Youn H.D. (2009). A nucleocytoplasmic malate dehydrogenase regulates p53 transcriptional ac-tivity in response to metabolic stress. Cell Death Differ..

[91] Qadri I., Iwahashi M., Simon F. (2002). Hepatitis C virus NS5A protein binds TBP and p53, inhibiting their DNA binding and p53 interactions with TBP and ERCC3. Biochim. Biophys. Acta (BBA) Mol. Cell Res..

[92] Liu R., Long Y., Liu R., Song G., Li Q., Yan H., Cui Z. (2022). Understanding the Function and Mechanism of Zebrafish Tmem39b in Regulating Cold Resistance. Int. J. Mol. Sci..

[93] Zheng D., Li C., Wang S., Cang Y., Song Y., Liu X., Li X., Mohan C., Wu T., Hu D. (2015). PSTK is a novel gene associated with early lung injury in Paraquat Poisoning. Life Sci..

[94] Esposito D., Crescenzi E., Sagar V., Loreni F., Russo A., Russo G. (2014). Human rpL3 plays a crucial role in cell response to nucleolar stress induced by 5-FU and L-OHP. Oncotarget.

[95] Abu-Odeh M., Salah Z., Herbel C., Hofmann T.G., Aqeilan R.I. (2014). WWOX, the common fragile site FRA16D gene product, regulates ATM activation and the DNA damage response. Proc. Natl. Acad. Sci. USA.

[96] Abu-Odeh M., Hereema N.A., Aqeilan R.I. (2015). WWOX modulates the ATR-mediated DNA damage checkpoint response. Oncotarget.

[97] Geyer C.B., Oatley J.M., Griswold M.D. (2017). Setting the Stage: The First Round of Spermatogenesis. The Biology of Mammalian Spermatogonia.

[98] La H.M., Mäkelä J.-A., Chan A.-L., Rossello F.J., Nefzger C.M., Legrand J.M.D., De Seram M., Polo J.M., Hobbs R.M. (2018). Identification of dynamic undifferentiated cell states within the male germline. Nat. Commun..

[99] Law N.C., Oatley M.J., Oatley J.M. (2019). Developmental kinetics and transcriptome dynamics of stem cell specification in the spermatogenic lineage. Nat. Commun..

[100] McNamara K.J. (2012). Heterochrony: The Evolution of Development. Evol. Educ. Outreach.

[101] Ramm S.A., Schärer L., Ehmcke J., Wistuba J. (2014). Sperm competition and the evolution of spermatogenesis. Mol. Hum. Reprod..

[102] Trumpp A., Essers M., Wilson A. (2010). Awakening dormant haematopoietic stem cells. Nat. Rev. Immunol..

[103] Lillie S.H., Pringle J.R. (1980). Reserve carbohydrate metabolism in Saccharomyces cerevisiae: Responses to nutrient limitation. J. Bacteriol..

[104] Otsuki L., Brand A.H. (2018). Cell cycle heterogeneity directs the timing of neural stem cell activation from quiescence. Science.

[105] Otsuki L., Brand A.H. (2020). Quiescent Neural Stem Cells for Brain Repair and Regeneration: Lessons from Model Systems. Trends Neurosci..

[106] Wong C., Kadekar P., Jurczak E., Roy R. (2023). Germline stem cell integrity and quiescence are controlled by an AMPK-dependent neuronal trafficking pathway. PLoS Genet..

[107] Kadekar P., Chaouni R., Clark E., Kazanets A., Roy R. (2018). Genome-wide surveys reveal polarity and cytoskeletal regulators mediate LKB1-associated germline stem cell quiescence. BMC Genom..

[108] Nguyen P.D., Gurevich D.B., Sonntag C., Hersey L., Alaei S., Nim H.T., Siegel A., Hall T.E., Rossello F.J., Boyd S.E. (2017). Muscle Stem Cells Undergo Extensive Clonal Drift during Tissue Growth via Meox1-Mediated Induction of G2 Cell-Cycle Arrest. Cell Stem Cell.

[109] Sutcu H.H., Ricchetti M. (2018). Loss of heterogeneity, quiescence, and differentiation in muscle stem cells. Stem Cell Investig..

[110] Montazid S., Bandyopadhyay S., Hart D.W., Gao N., Johnson B., Thrumurthy S.G., Penn D.J., Wernisch B., Bansal M., Altrock P.M. (2023). Adult stem cell activity in naked mole rats for long-term tissue maintenance. Nat. Commun..

[111] Buzgariu W., Crescenzi M., Galliot B. (2014). Robust G2 pausing of adult stem cells in Hydra. Differentiation.

[112] Baudat F., Buard J., Grey C., Fledel-Alon A., Ober C., Przeworski M., Coop G., de Massy B. (2010). PRDM9 is a major determinant of meiotic recombination hotspots in humans and mice. Science.

[113] Suzuki A., Niimi Y., Shinmyozu K., Zhou Z., Kiso M., Saga Y. (2016). Dead end1 is an essential partner of NANOS2 for selective binding of target RNAs in male germ cell devel-opment. EMBO Rep..

[114] Yoshida S. (2018). Open niche regulation of mouse spermatogenic stem cells. Dev. Growth Differ..

[115] Irie N., Weinberger L., Tang W.W., Kobayashi T., Viukov S., Manor Y.S., Dietmann S., Hanna J.H., Surani M.A. (2014). SOX17 Is a Critical Specifier of Human Primordial Germ Cell Fate. Cell.

[116] Mitsunaga S., Shioda T. (2018). Evolutionarily diverse mechanisms of germline specification among mammals: What about us?. Stem Cell Investig..

[117] Tacutu R., Thornton D., Johnson E., Budovsky A., Barardo D., Craig T., Diana E., Lehmann G., Toren D., Wang J. (2017). Human Ageing Genomic Resources: New and updated databases. Nucleic Acids Res..

[118] Ouyang J.F., Kamaraj U.S., Cao E.Y., Rackham O.J.L. (2021). ShinyCell: Simple and sharable visualization of single-cell gene expression data. Bioinformatics.

