# Supplementary material for: Adult Human, but Not Rodent, Spermatogonial Stem Cells Retain States with a Foetal-like Signature
Source: Cells. 2024 Apr 24;13(9):742. doi: 10.3390/cells13090742 (PMC11083513; doi:10.3390/cells13090742)
Supplement: Supplementary file 1 [file cells-13-00742-s001.zip › cells-2947143-supplementary/figure_s13.pdf]

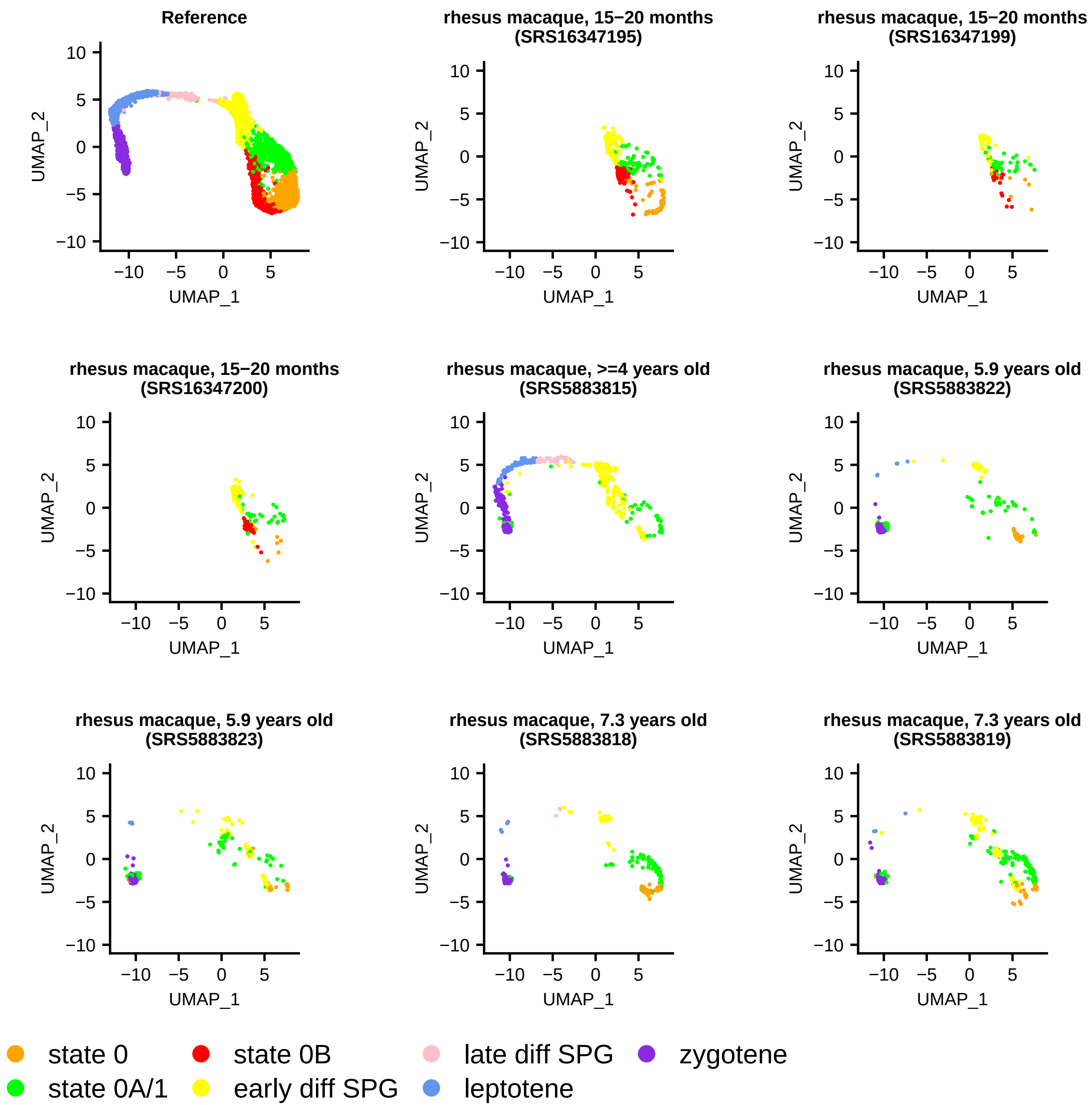
Reference
rhesus macaque, 15−20 months (SRS16347195)
rhesus macaque, 15−20 months (SRS16347199)
rhesus macaque, 15−20 months (SRS16347200)
rhesus macaque, >=4 years old (SRS5883815)
rhesus macaque, 5.9 years old (SRS5883822)
rhesus macaque, 5.9 years old (SRS5883823)
rhesus macaque, 7.3 years old (SRS5883818)
rhesus macaque, 7.3 years old (SRS5883819)
UMAP_1
UMAP_2
state 0
state 0A/1
state 0B
early diff SPG
late diff SPG
leptotene
zygotene
