# Supplementary material for: Adult Human, but Not Rodent, Spermatogonial Stem Cells Retain States with a Foetal-like Signature
Source: Cells. 2024 Apr 24;13(9):742. doi: 10.3390/cells13090742 (PMC11083513; doi:10.3390/cells13090742)
Supplement: Supplementary file 1 [file cells-13-00742-s001.zip › cells-2947143-supplementary/figure_s15.pdf]

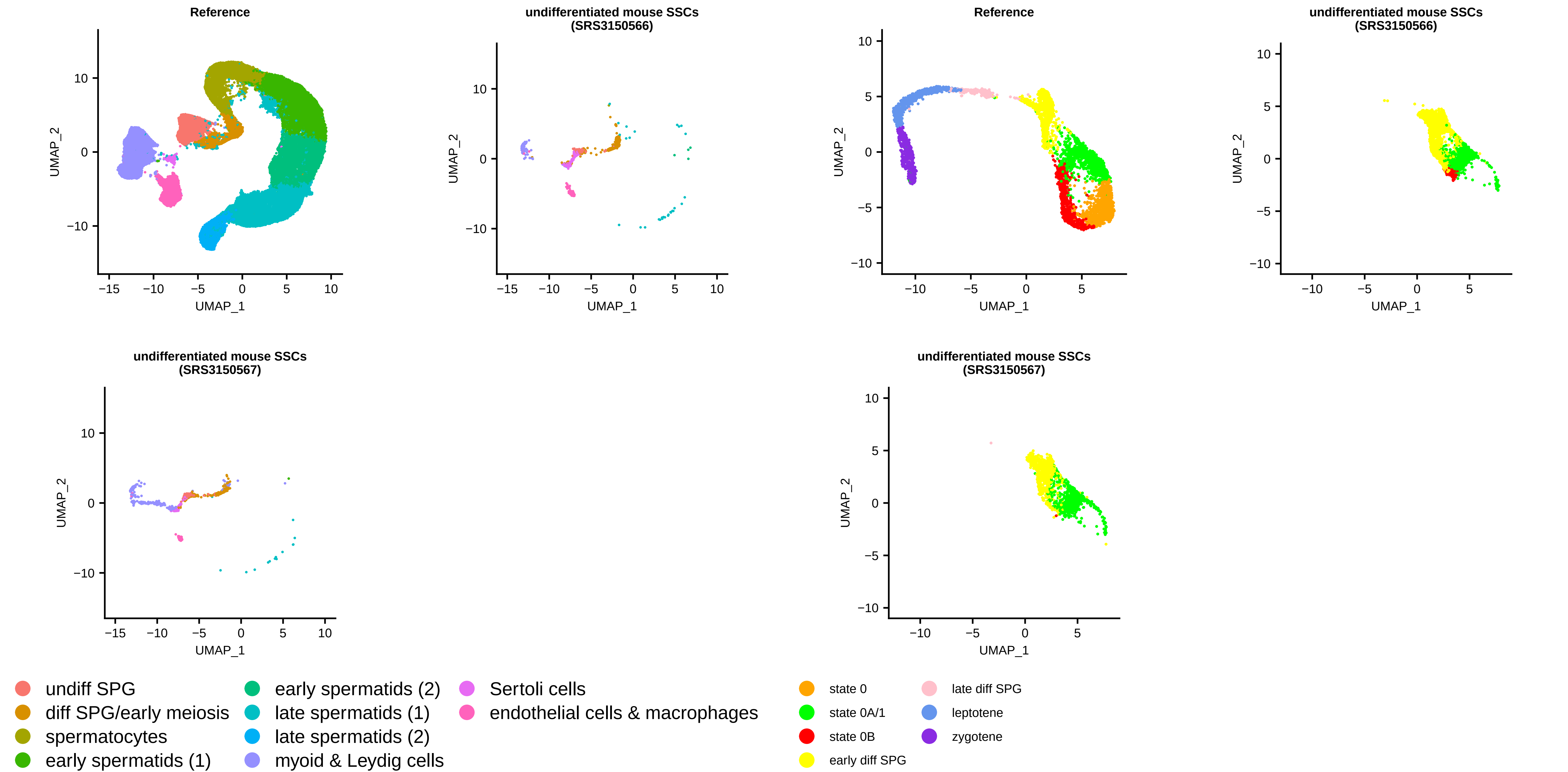

Reference
undifferentiated mouse SSCs (SRS3150566)
undifferentiated mouse SSCs (SRS3150567)
UMAP_1
UMAP_2
undiff SPG
diff SPG/early meiosis
spermatocytes
early spermatids (1)
early spermatids (2)
late spermatids (1)
late spermatids (2)
myoid & Leydig cells
Sertoli cells
endothelial cells & macrophages
state 0
state 0A/1
state 0B
early diff SPG
late diff SPG
leptotene
zygotene
