# Supplementary material for: Adult Human, but Not Rodent, Spermatogonial Stem Cells Retain States with a Foetal-like Signature
Source: Cells. 2024 Apr 24;13(9):742. doi: 10.3390/cells13090742 (PMC11083513; doi:10.3390/cells13090742)
Supplement: Supplementary file 1 [file cells-13-00742-s001.zip › cells-2947143-supplementary/figure_s16.pdf]

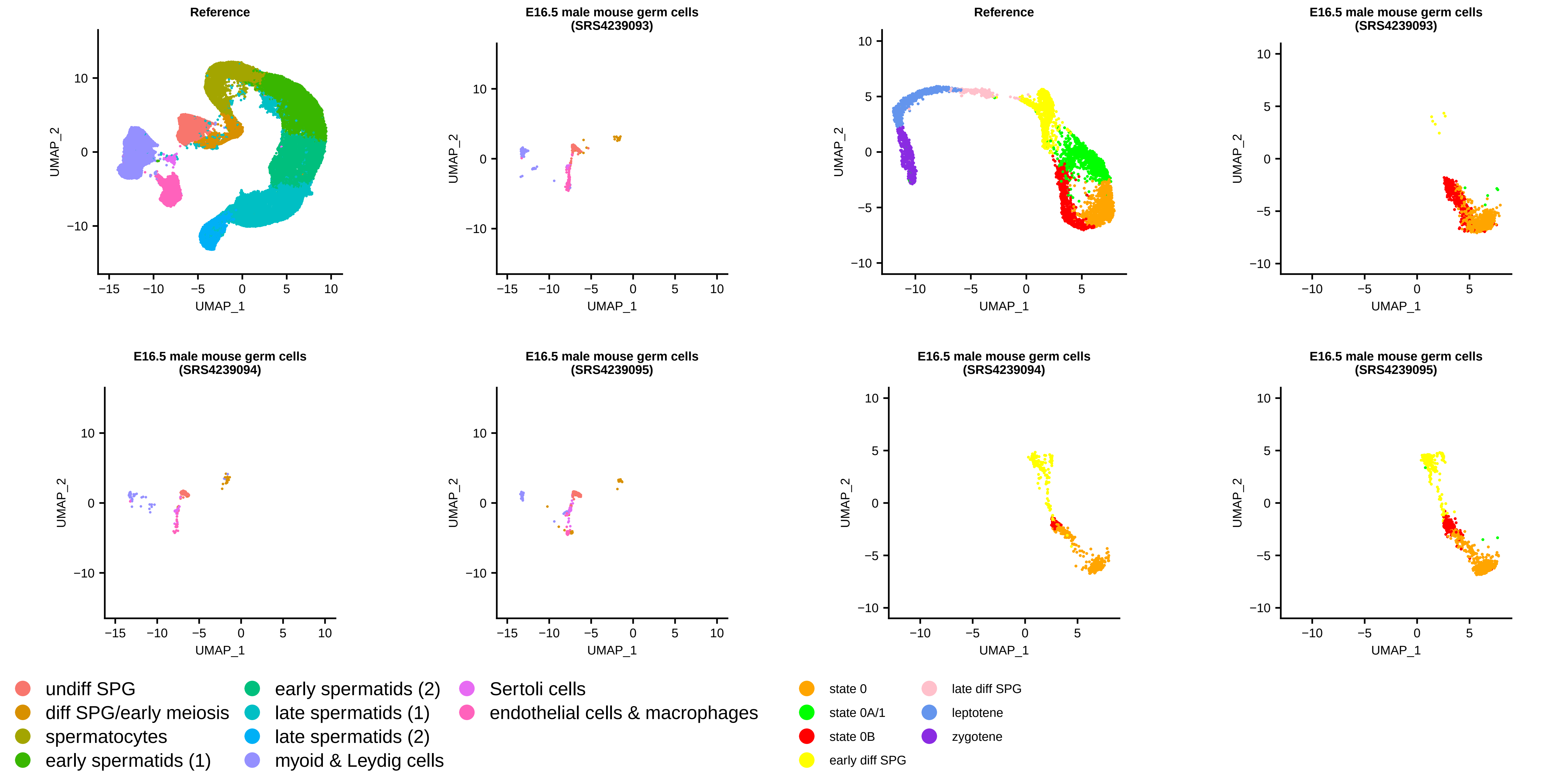

Reference
E16.5 male mouse germ cells (SRS4239093)
Reference
E16.5 male mouse germ cells (SRS4239093)
E16.5 male mouse germ cells (SRS4239094)
E16.5 male mouse germ cells (SRS4239095)
E16.5 male mouse germ cells (SRS4239094)
E16.5 male mouse germ cells (SRS4239095)
UMAP_1
UMAP_2
undiff SPG
diff SPG/early meiosis
spermatocytes
early spermatids (1)
early spermatids (2)
late spermatids (1)
late spermatids (2)
myoid & Leydig cells
Sertoli cells
endothelial cells & macrophages
state 0
state 0A/1
state 0B
early diff SPG
late diff SPG
leptotene
zygotene
