# Supplementary figures and images for: Adult Human, but Not Rodent, Spermatogonial Stem Cells Retain States with a Foetal-like Signature
Source: Cells. 2024 Apr 24;13(9):742. doi: 10.3390/cells13090742 (PMC11083513; doi:10.3390/cells13090742)

Study of origin

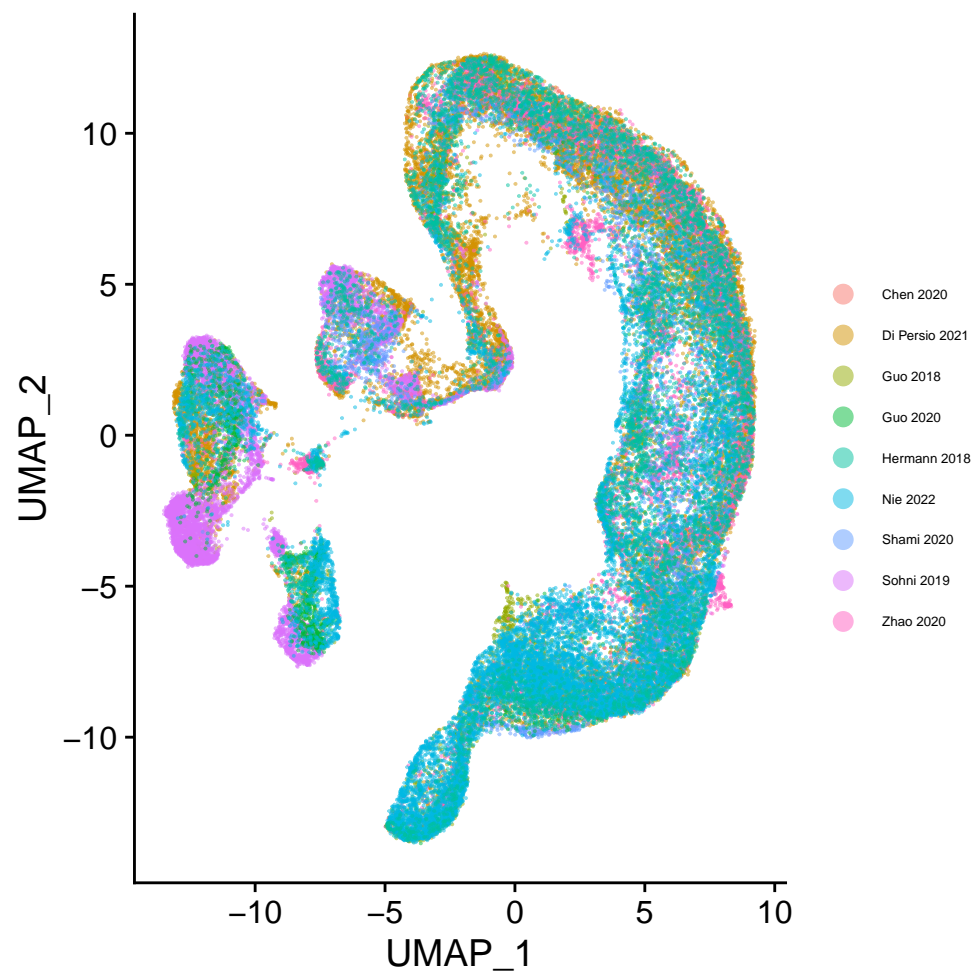

Age of donor

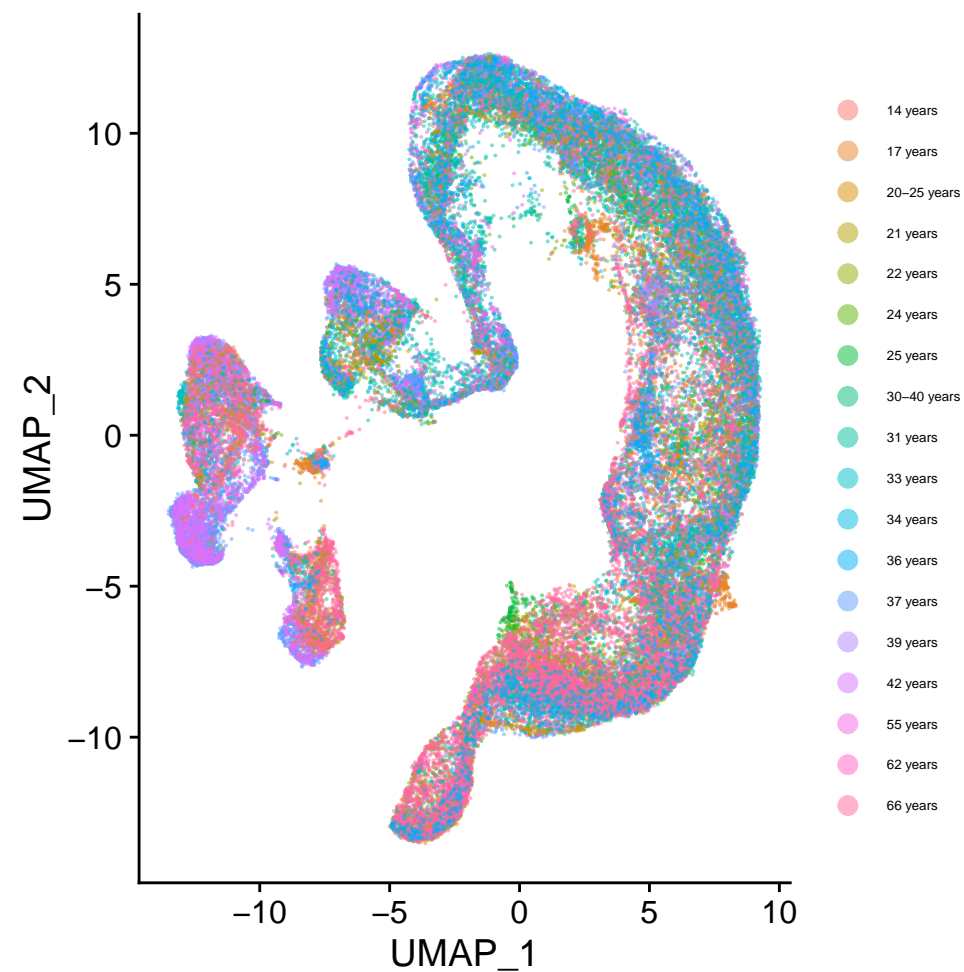

Sample accession

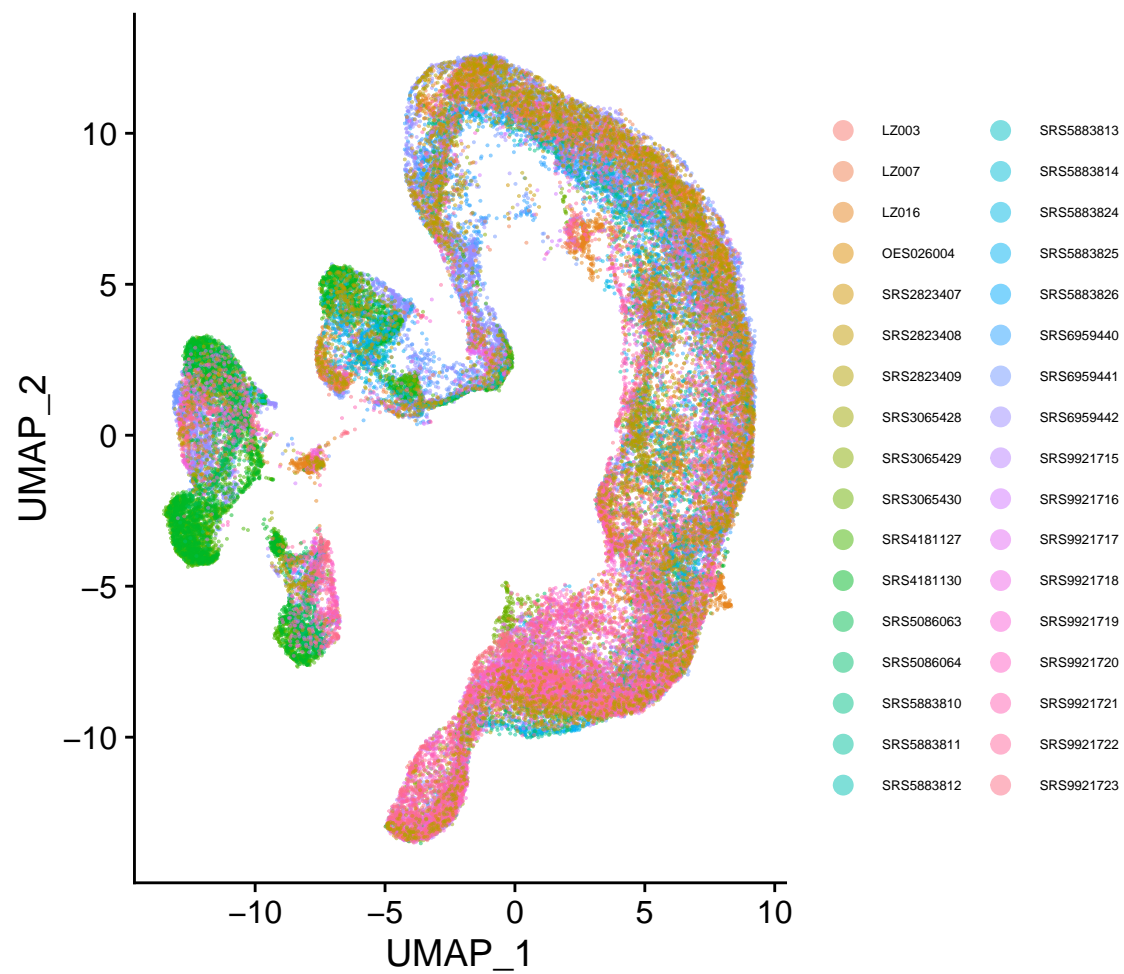

Phase of cell cycle

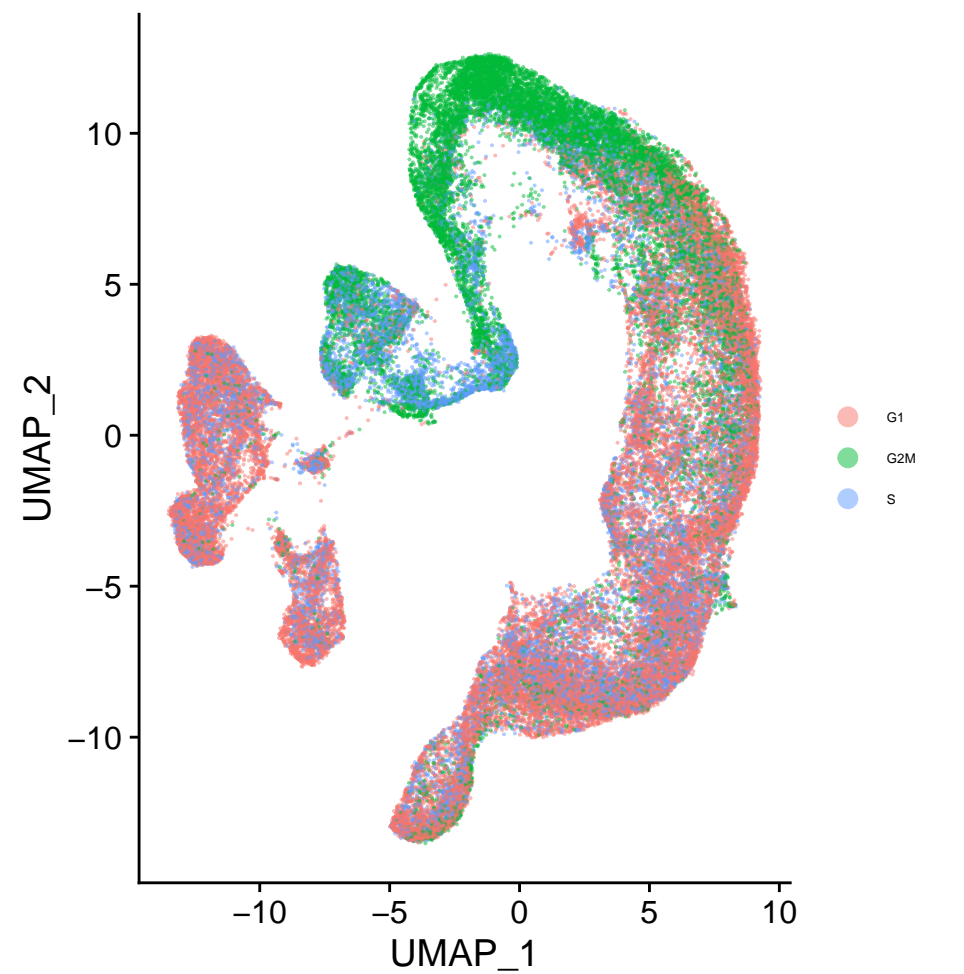

Supplement: Supplementary file 1 [file cells-13-00742-s001.zip › cells-2947143-supplementary/figure_s1.pdf]

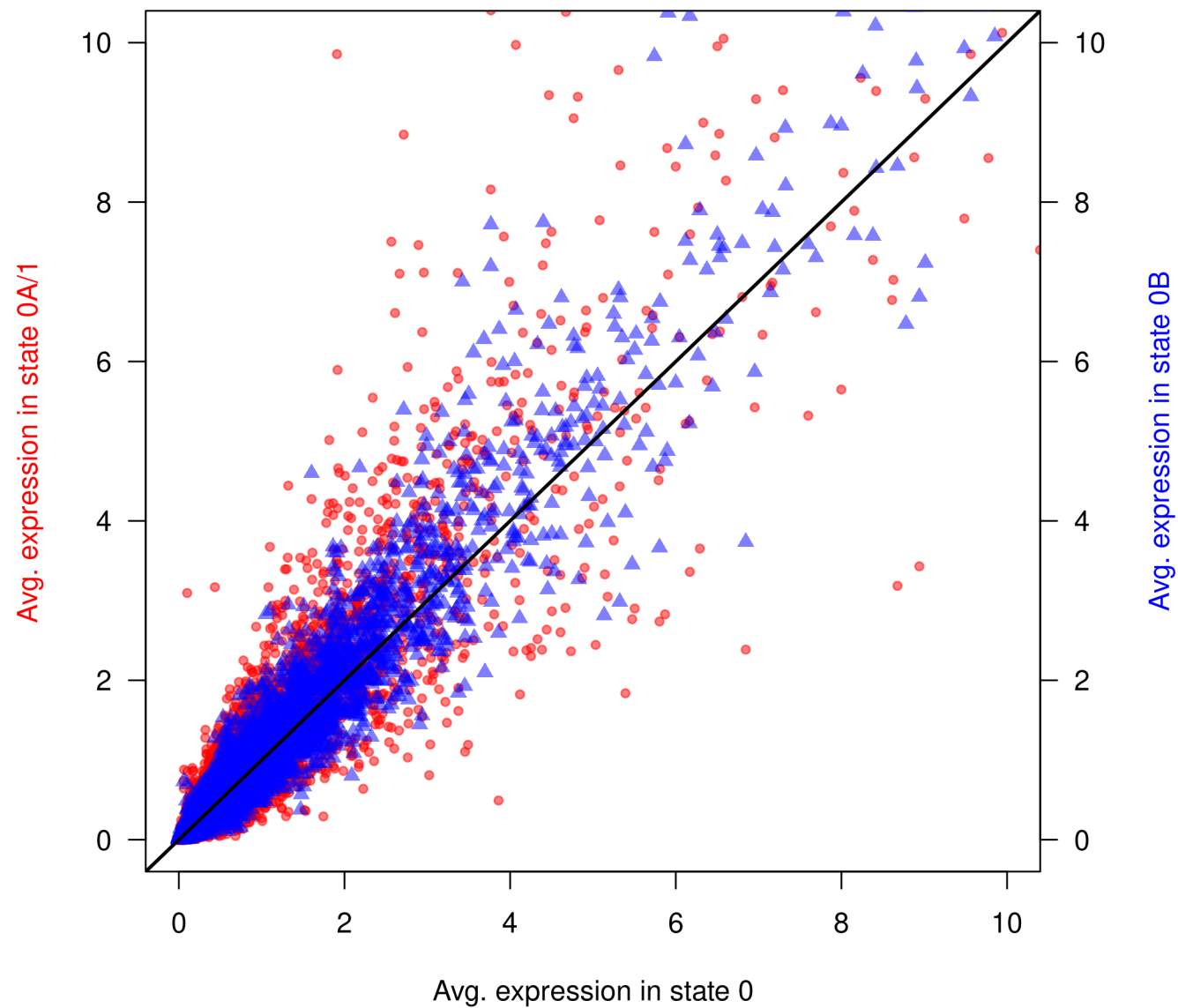

Supplement: Supplementary file 1 [file cells-13-00742-s001.zip › cells-2947143-supplementary/figure_s10.pdf]

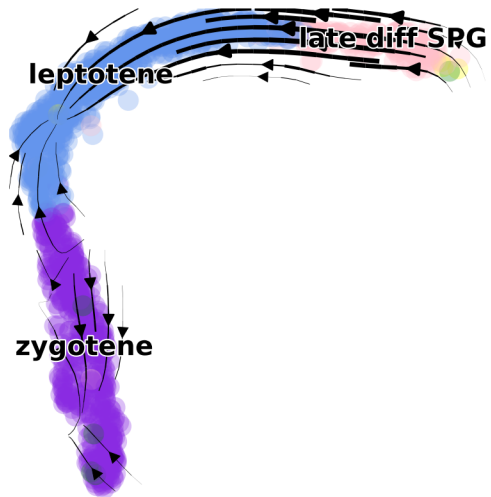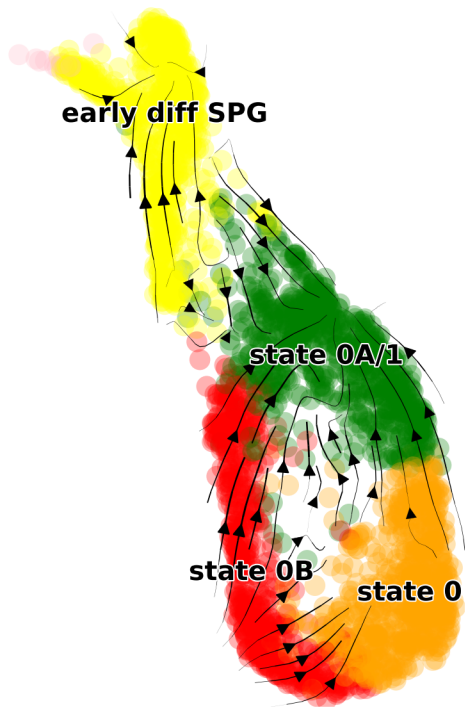

Supplement: Supplementary file 1 [file cells-13-00742-s001.zip › cells-2947143-supplementary/figure_s11.pdf]

A

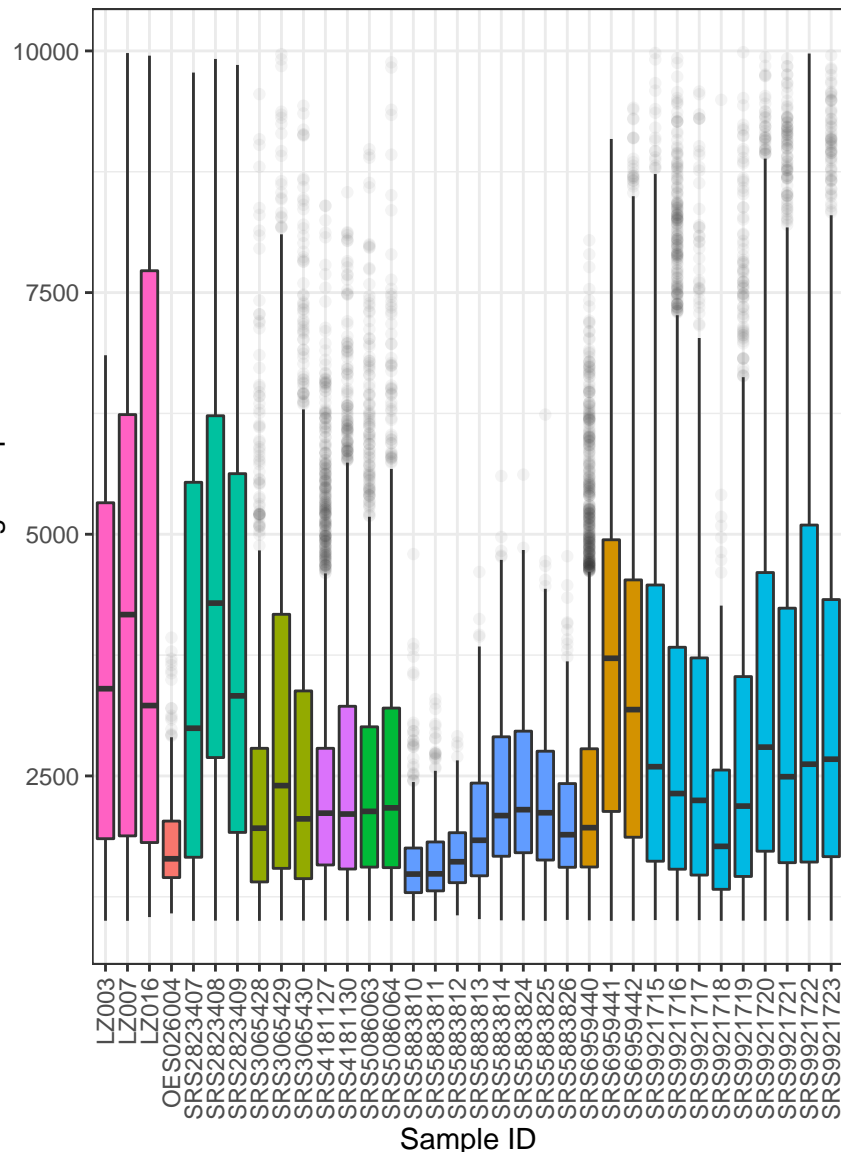

B

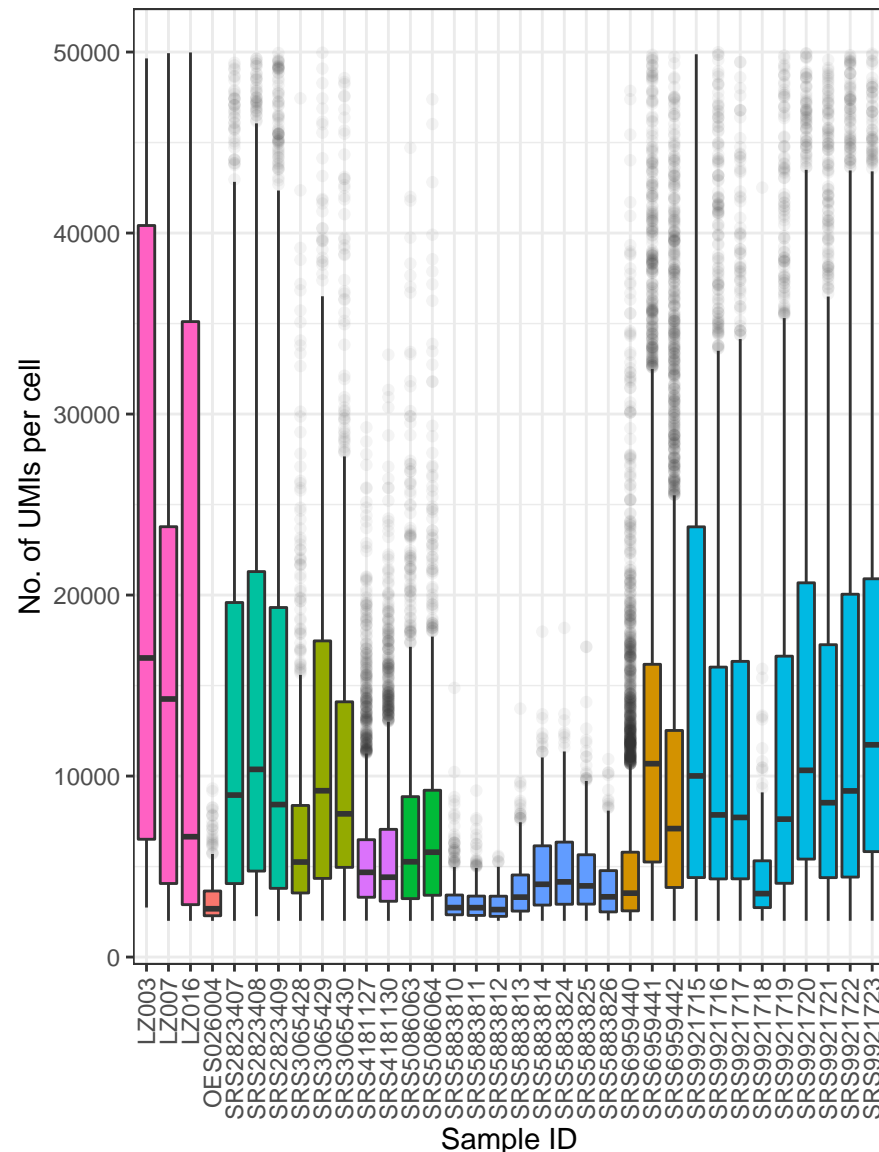

C

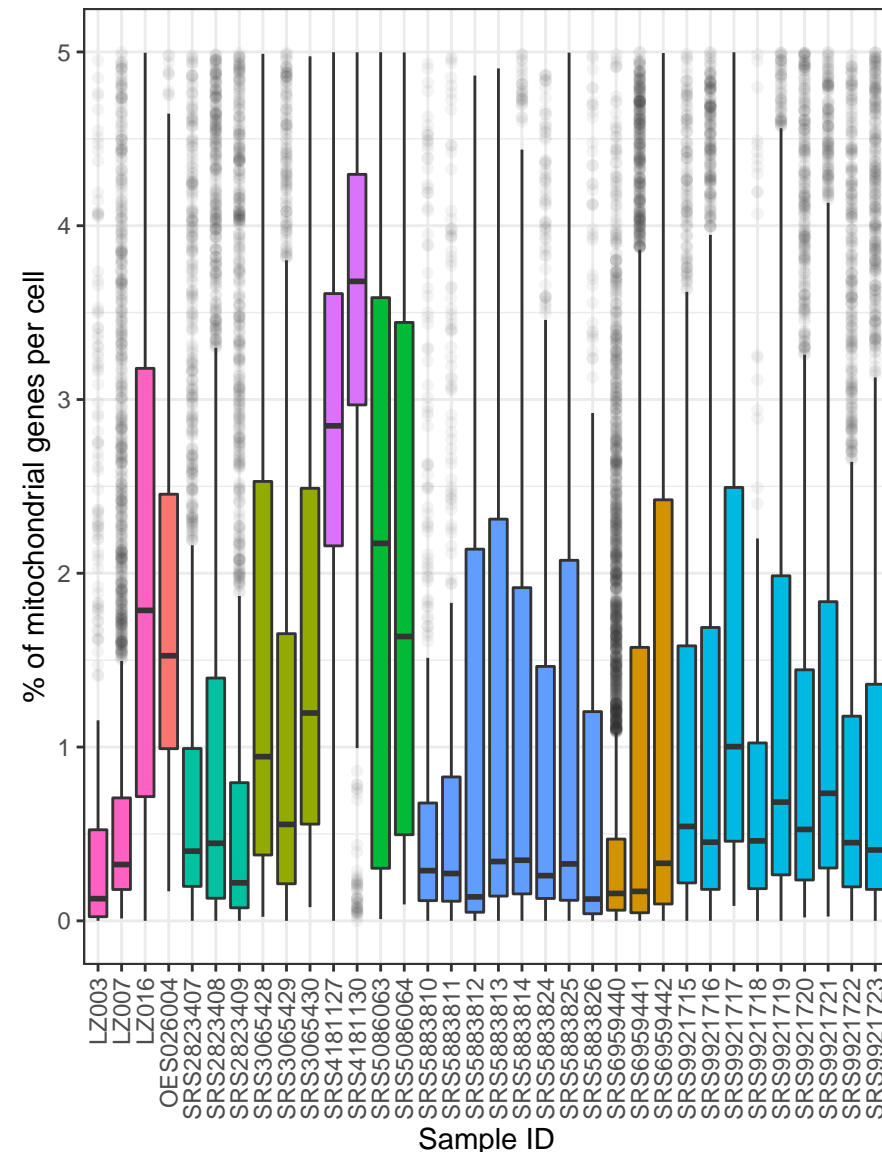

source

|                |          |              |            |           |
|----------------|----------|--------------|------------|-----------|
| Chen 2020      | Guo 2018 | Hermann 2018 | Shami 2020 | Zhao 2020 |
| Di Persio 2021 | Guo 2020 | Nie 2022     | Sohni 2019 |           |

Supplement: Supplementary file 1 [file cells-13-00742-s001.zip › cells-2947143-supplementary/figure_s2.pdf]

**Study of origin**

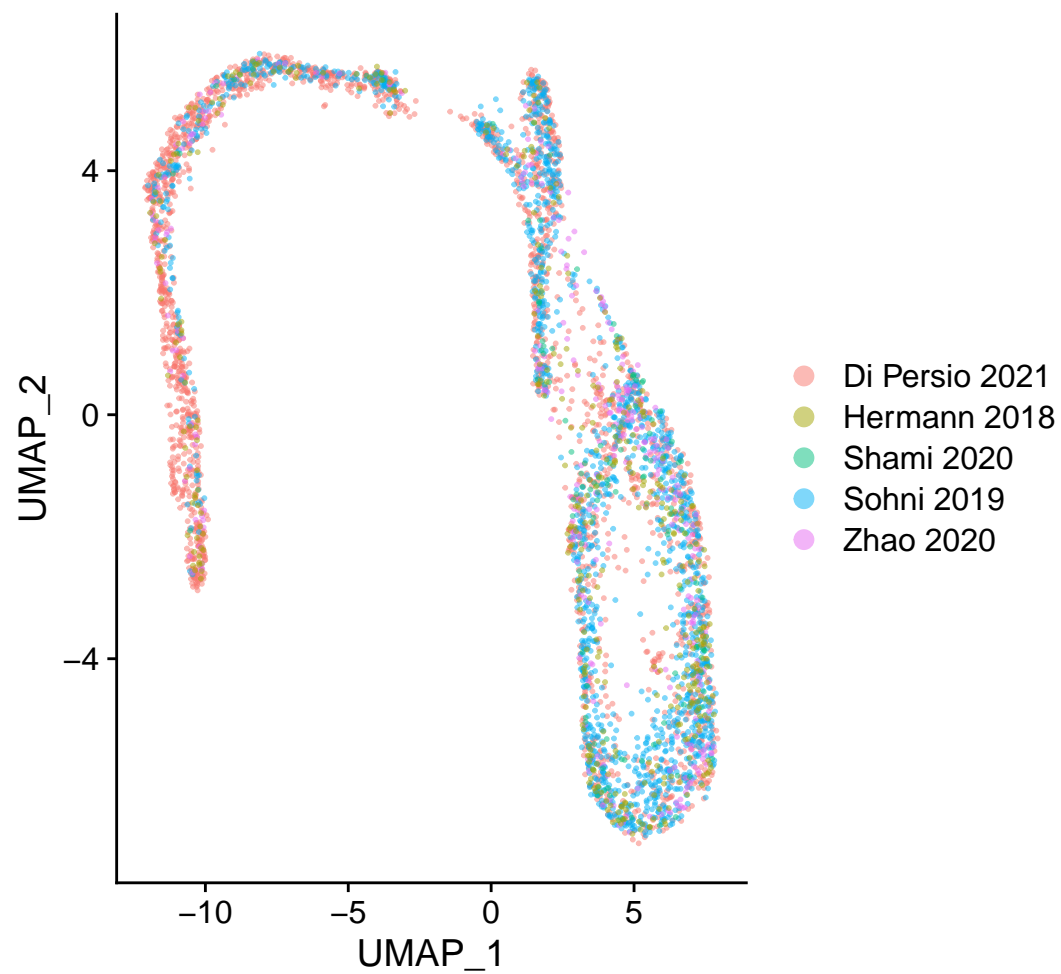

**Age of donor**

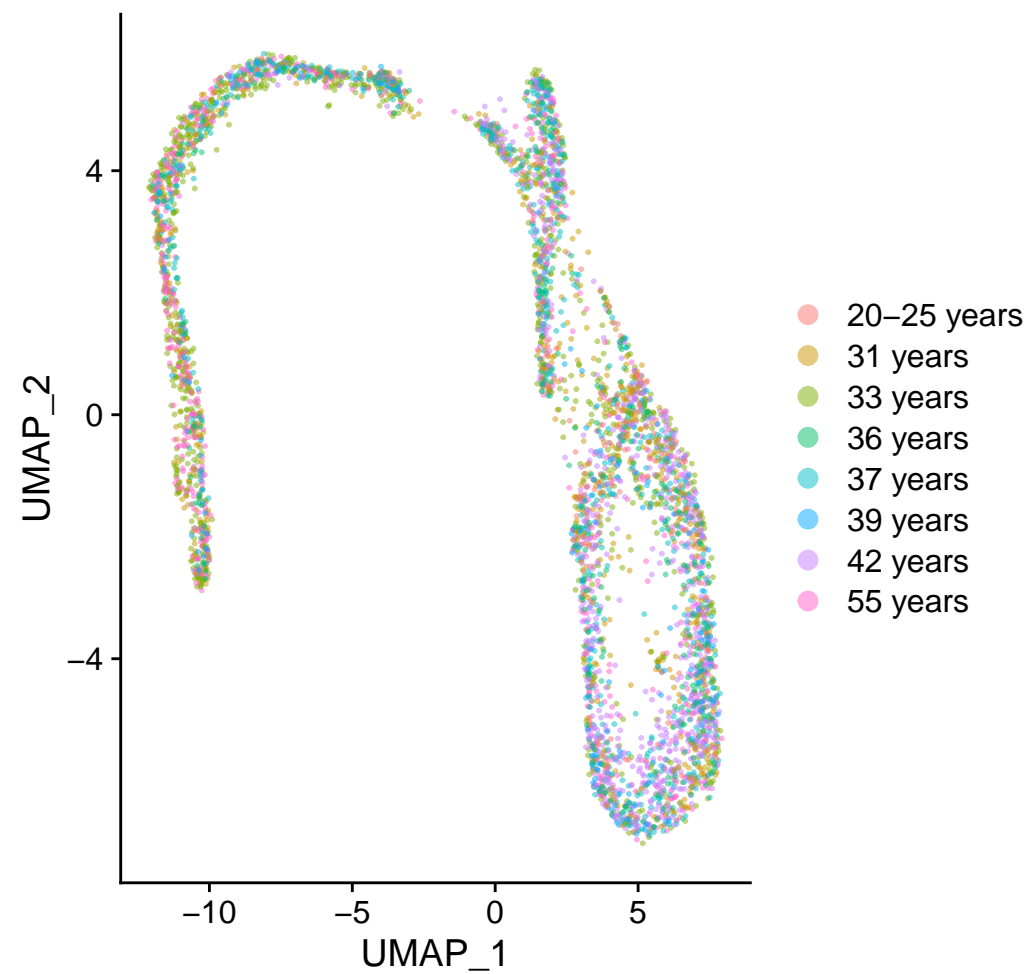

**Sample accession**

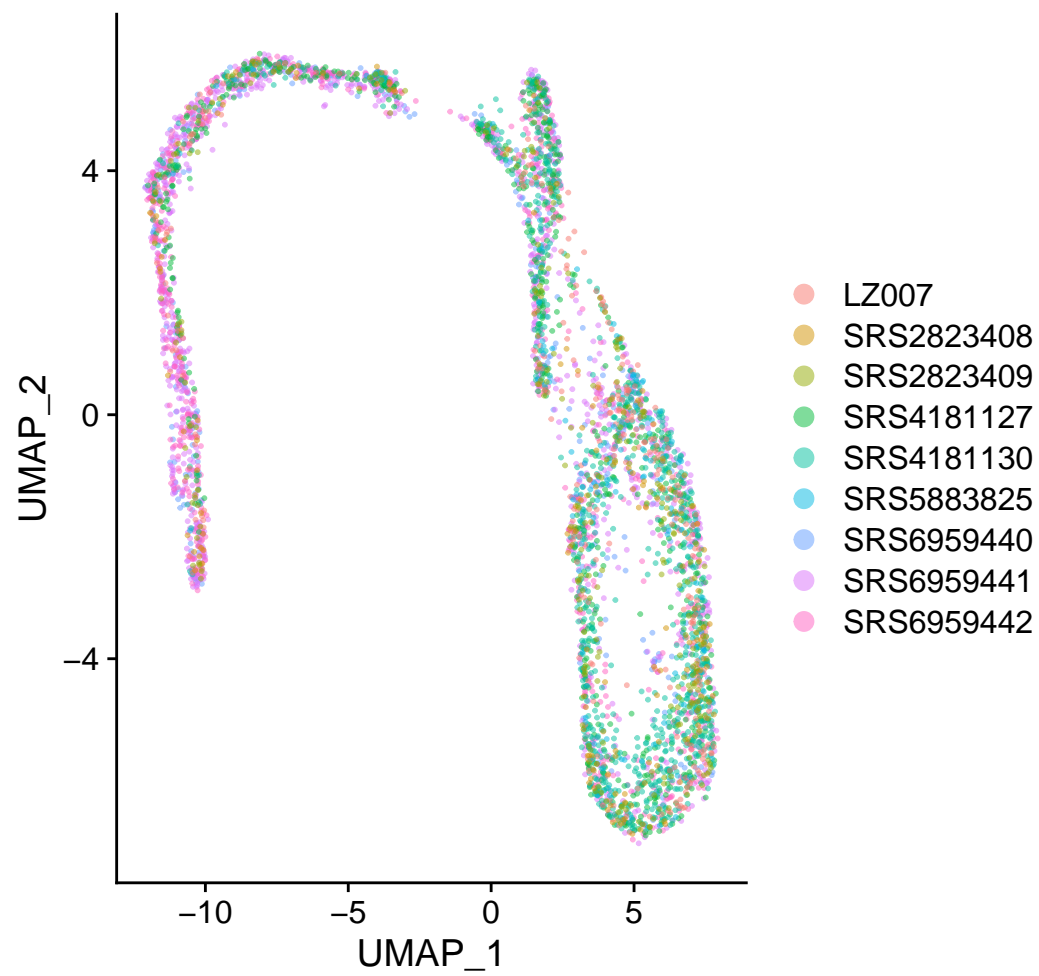

**Phase of cell cycle**

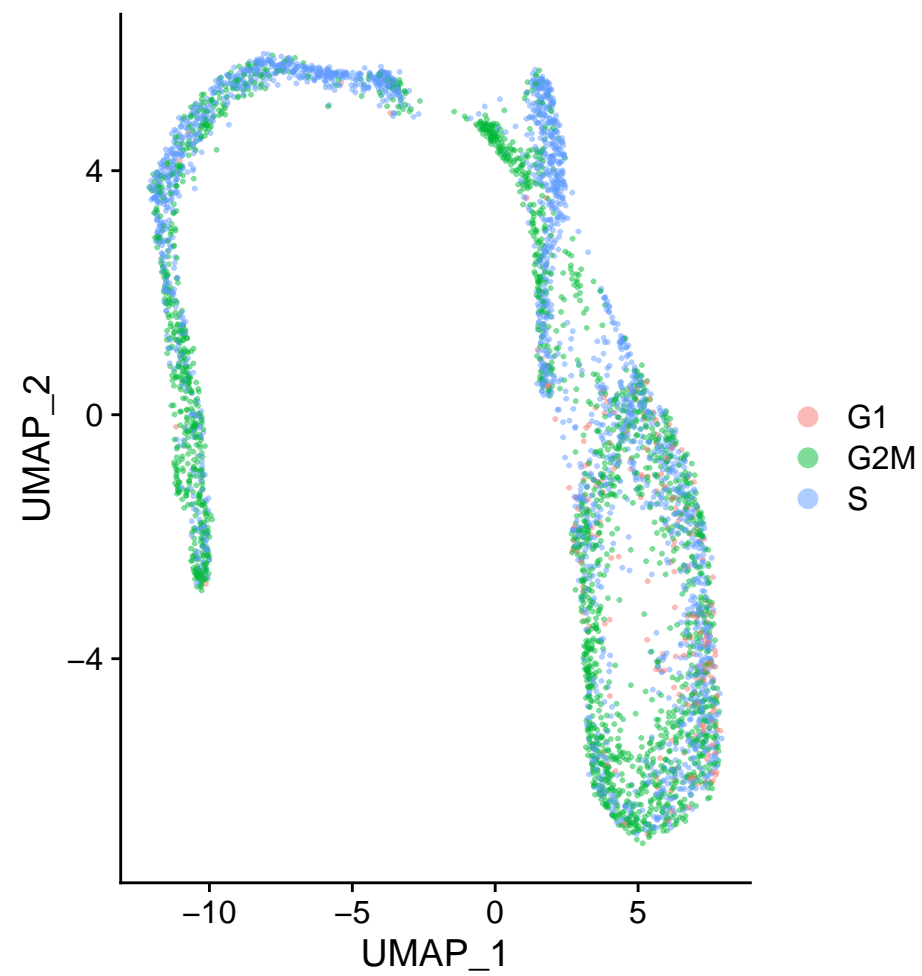

Supplement: Supplementary file 1 [file cells-13-00742-s001.zip › cells-2947143-supplementary/figure_s3.pdf]

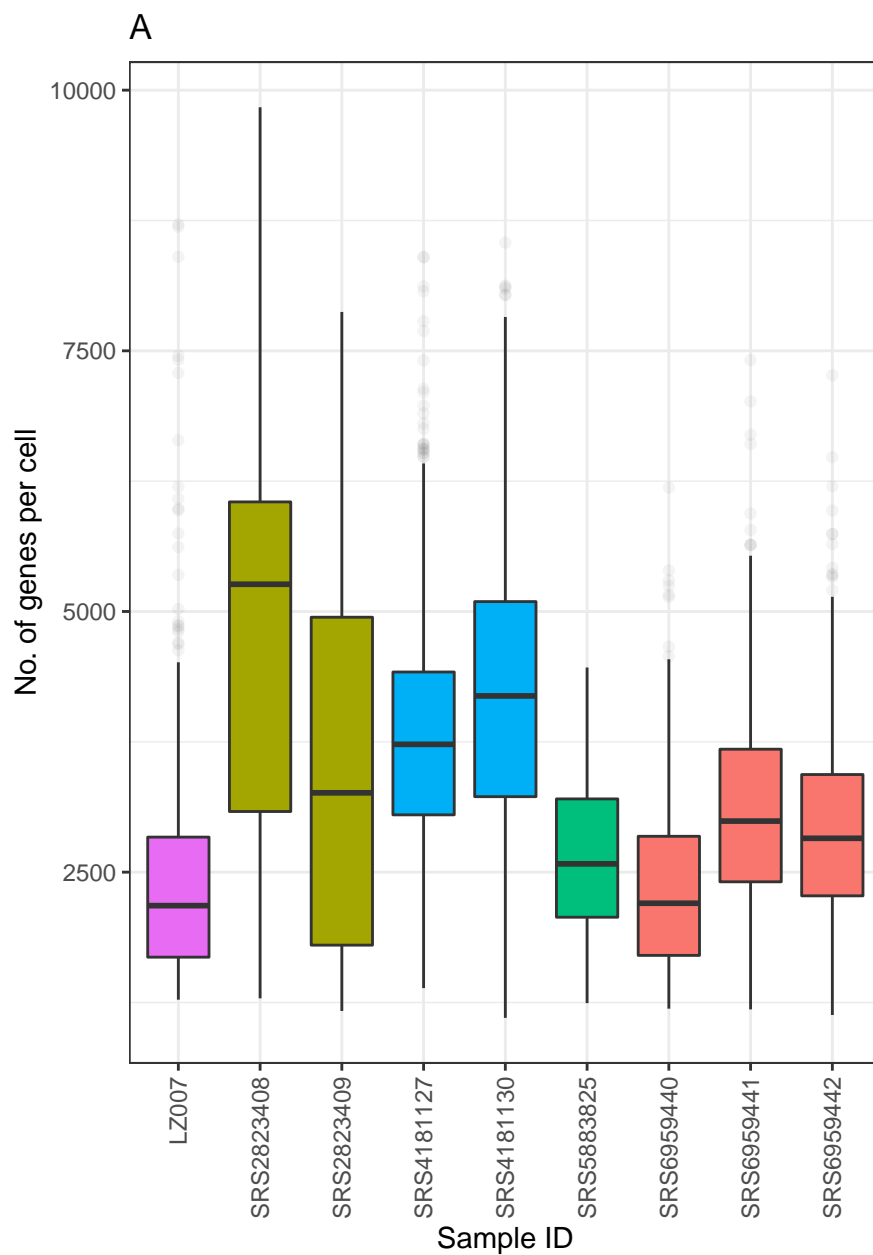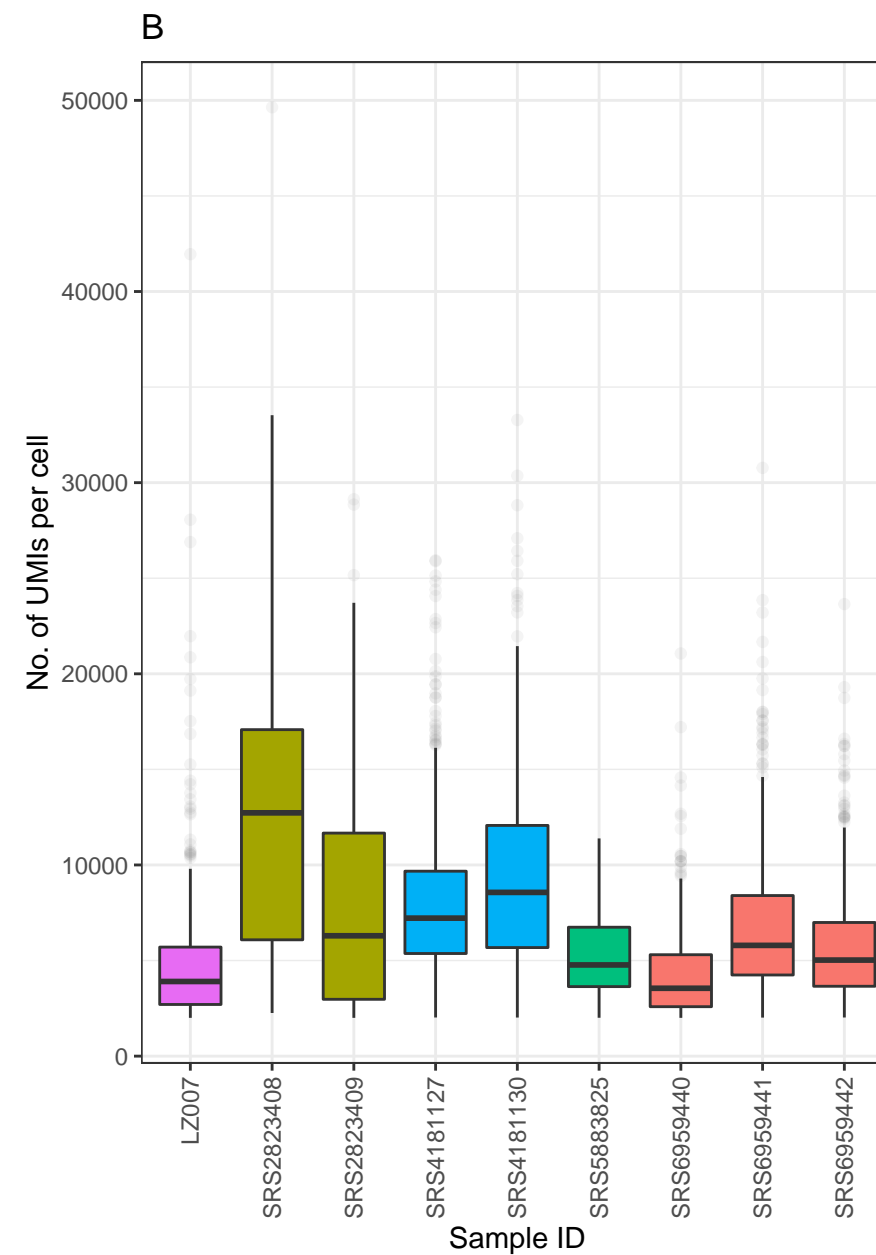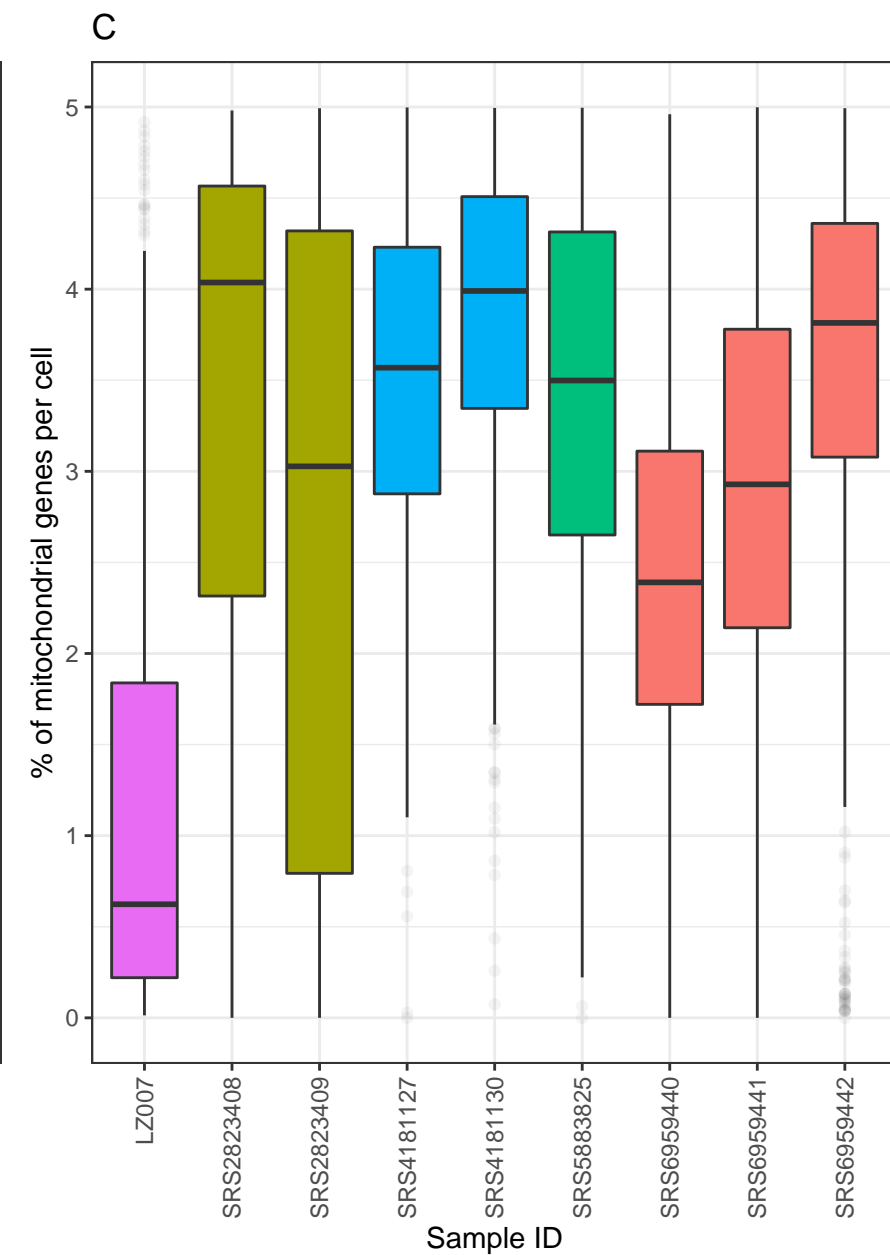

source Di Persio 2021 Hermann 2018 Shami 2020 Sohni 2019 Zhao 2020

Supplement: Supplementary file 1 [file cells-13-00742-s001.zip › cells-2947143-supplementary/figure_s4.pdf]

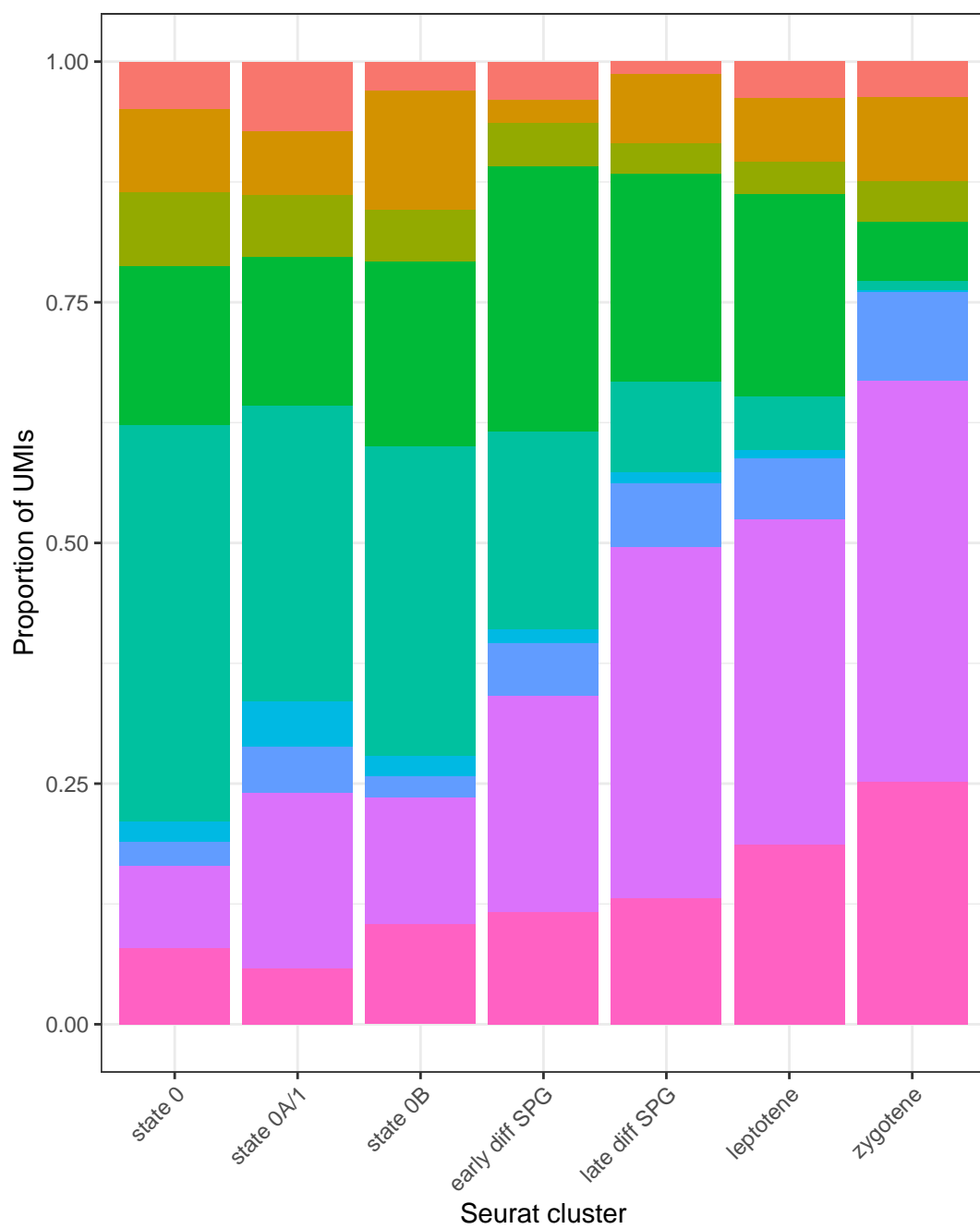

Sample ID

- LZ007
- SRS2823408
- SRS2823409
- SRS4181127
- SRS4181130
- SRS5883825
- SRS6959440
- SRS6959441
- SRS6959442

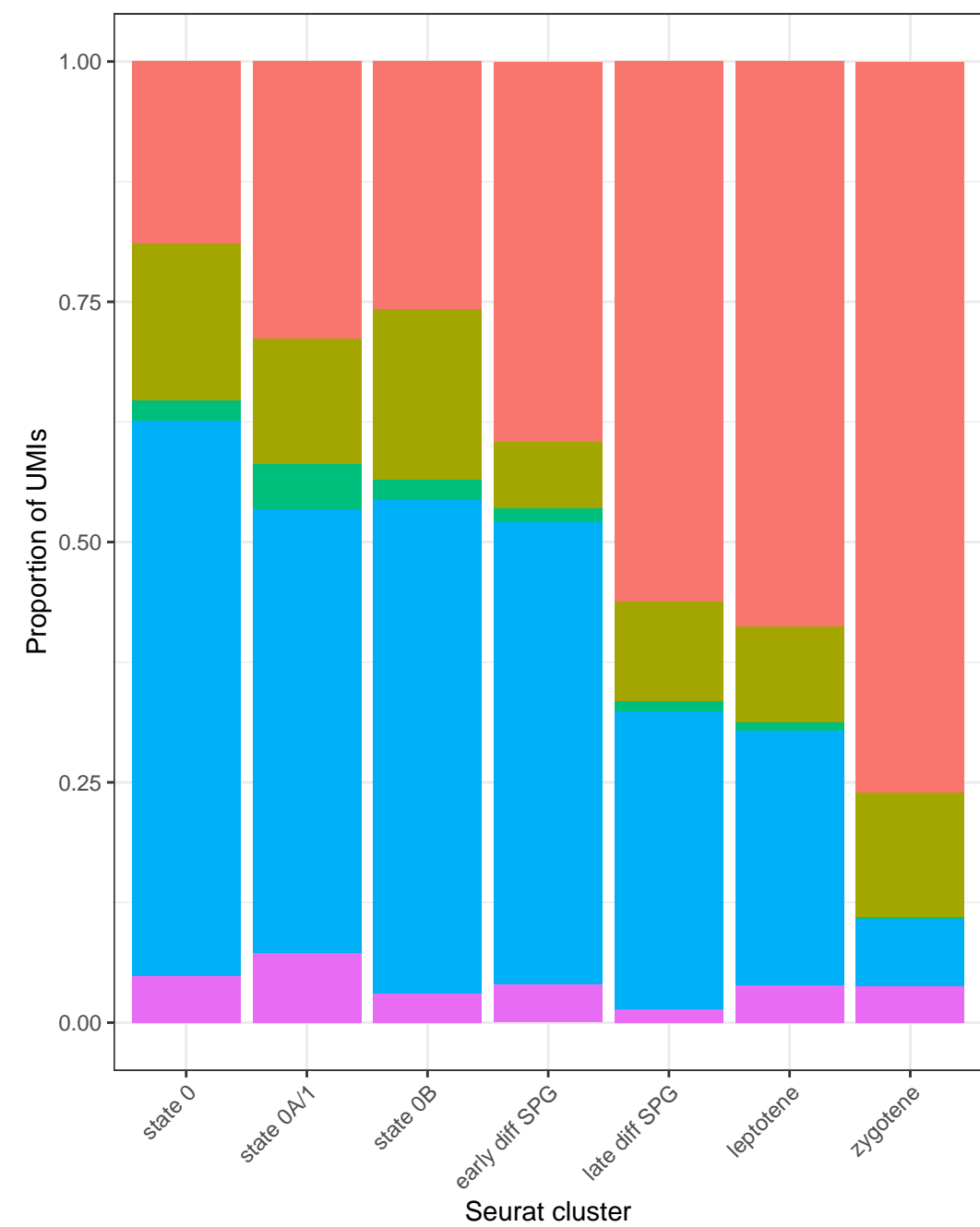

Sample ID

- Di Persio 2021
- Hermann 2018
- Shami 2020
- Sohni 2019
- Zhao 2020

Supplement: Supplementary file 1 [file cells-13-00742-s001.zip › cells-2947143-supplementary/figure_s5.pdf]

**EGR4**

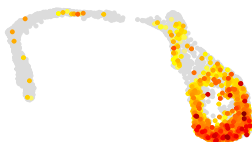

**PIWIL4**

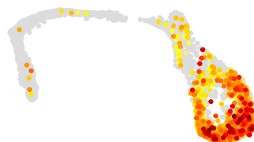

**UTF1**

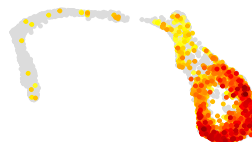

**TCF3**

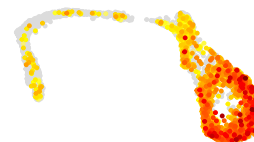

**TSPAN33**

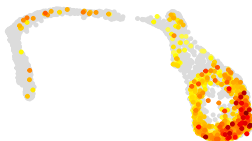

**FGFR3**

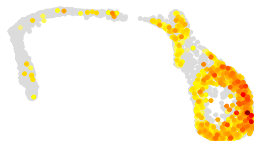

**ID4**

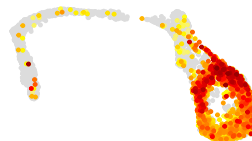

**GPX1**

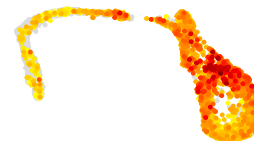

**GFRA1**

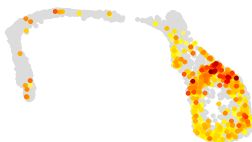

**NANOS3**

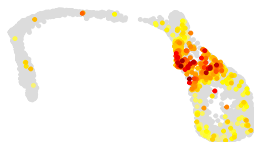

**NANOS2**

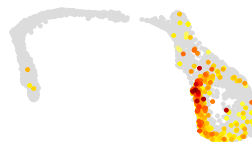

**MAGEA4**

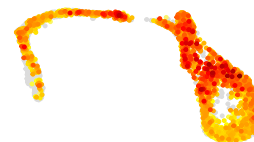

**KIT**

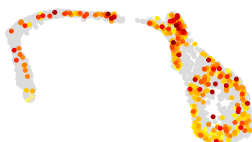

**MKI67**

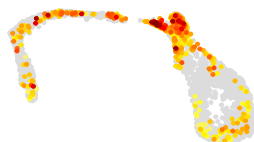

**DMRT1**

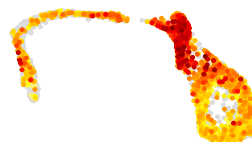

**STRA8**

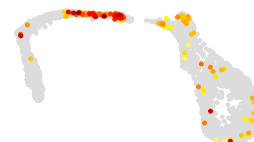

**MEIOSIN**

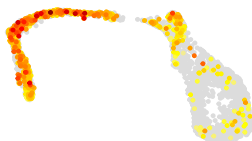

**TKTL1**

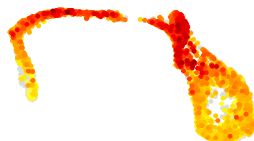

**SYCP3**

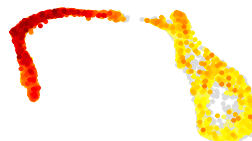

**SPO11**

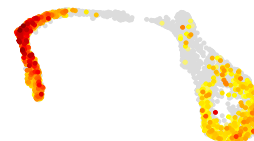

Supplement: Supplementary file 1 [file cells-13-00742-s001.zip › cells-2947143-supplementary/figure_s7.pdf]

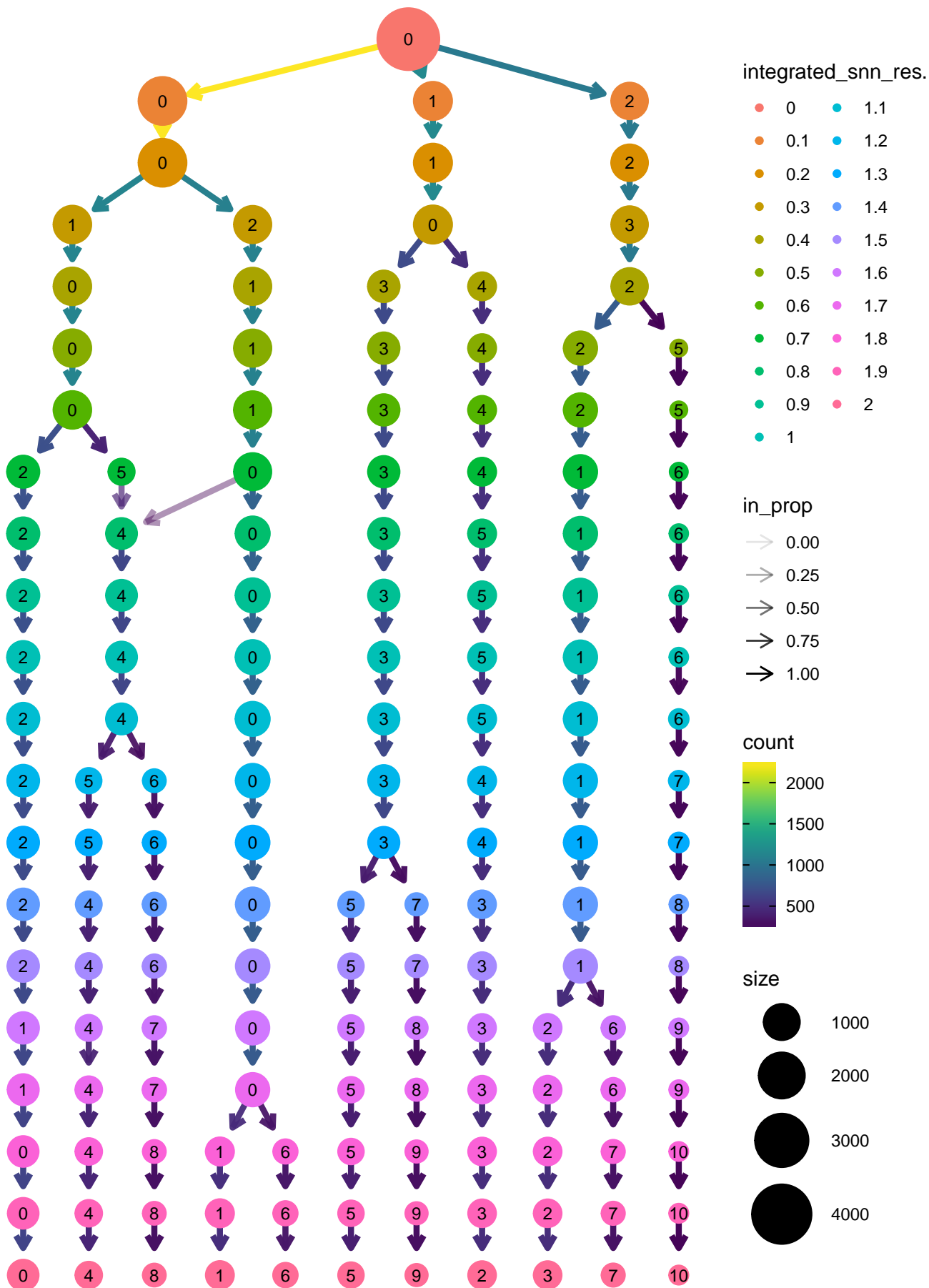

Supplement: Supplementary file 1 [file cells-13-00742-s001.zip › cells-2947143-supplementary/figure_s8.pdf]

**A**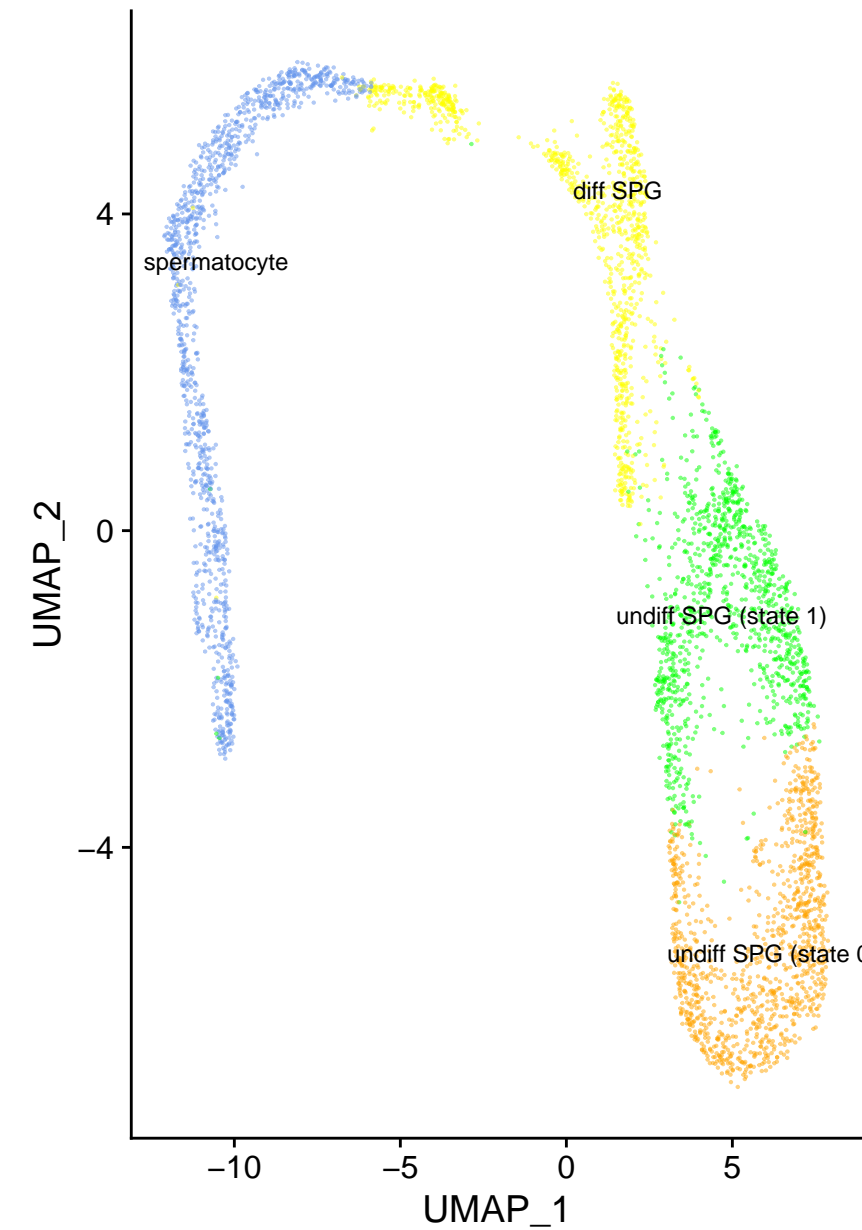**B**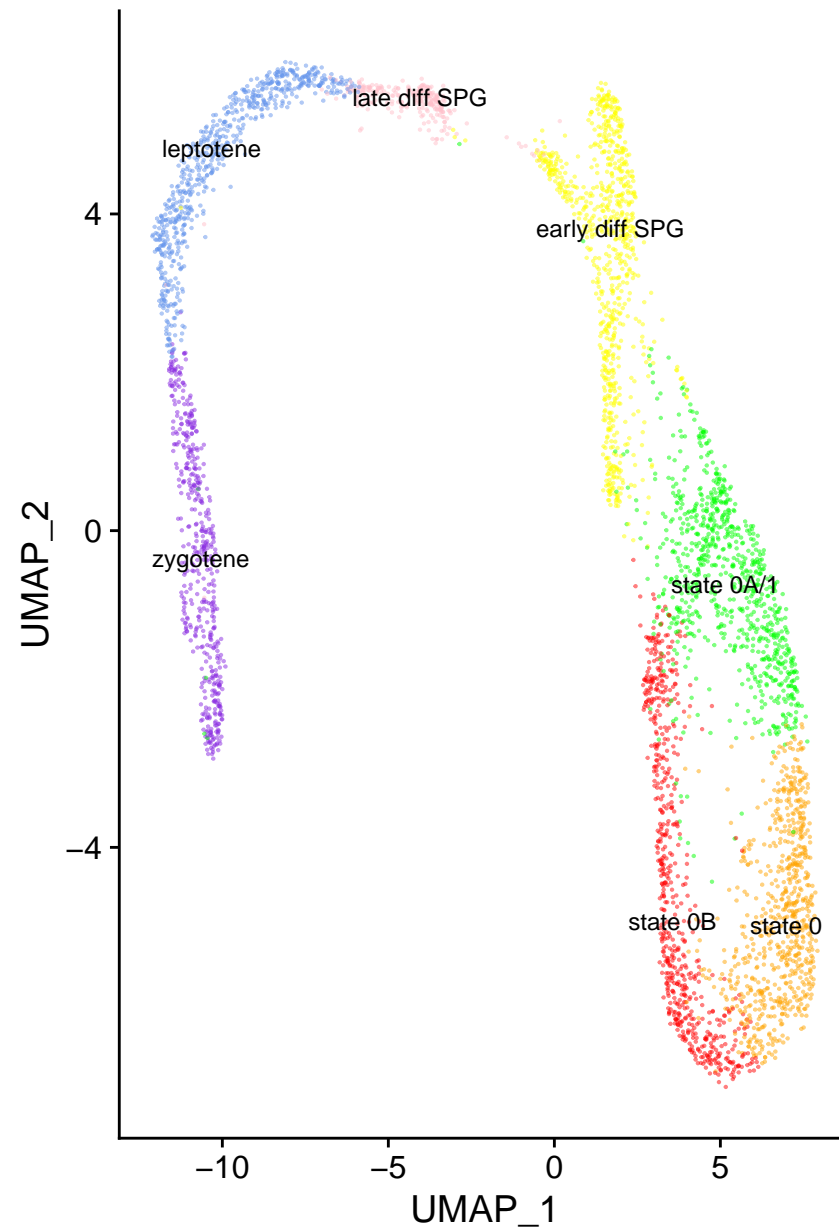**C**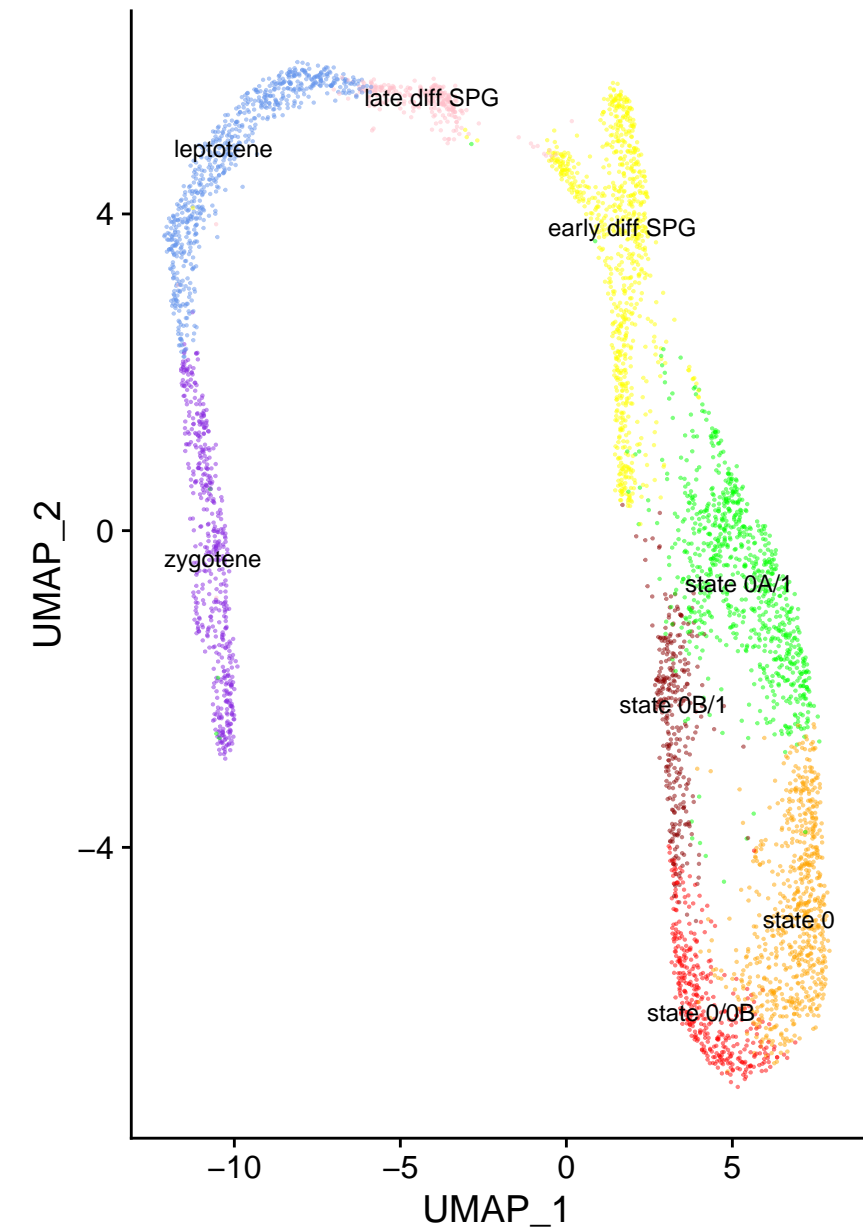

Supplement: Supplementary file 1 [file cells-13-00742-s001.zip › cells-2947143-supplementary/figure_s9.pdf]
